# SFARP: a multi-layered real-time security framework for hybrid ARP and DDoS attack defense in SD-IoT networks

**DOI:** 10.1038/s41598-025-28830-9

**Published:** 2025-12-09

**Authors:** Ameer El-Sayed, Hagar Ramadan, Ehab R. Mohamed, Osama M. Elkomy

**Affiliations:** Department of Information Technology, Faculty of Computers and Informatics, Zagazig University, Zagazig, 44511 Egypt

**Keywords:** IoT, SDN, DDoS detection, ARP spoofing, P4 language, Ensemble learning, Engineering, Mathematics and computing

## Abstract

The rapid expansion of Software-Defined Internet of Things (SD-IoT) networks has amplified both scalability and vulnerability, exposing them to increasingly sophisticated multi-vector attacks such as flooding-based Distributed Denial-of-Service (DDoS), Address Resolution Protocol (ARP) spoofing, DNS spoofing, and MAC flooding. These threats exploit static control planes and centralized architectures, overwhelming controllers and bypassing threshold-based defenses through adaptive, sequential, and hybrid behaviors. To address these challenges, we propose SFARP, a multi-layered real-time security framework tailored for SD-IoT environments. SFARP integrates three coordinated modules: (1) the Dynamic Flow Analysis Module (DFAM), which leverages P4-programmed switches to extract fine-grained traffic and ARP-level features; (2) the Adaptive Dynamic Flow Detection System (ADFDS), which employs an ensemble of machine learning classifiers to detect anomalies across hybrid and multi-vector attack scenarios; and (3) the Distributed Adaptive Mitigation System (DAMS), which deploys adaptive countermeasures across a multi-controller SDN topology. In addition, we extend the evaluation to multi-vector attacks (ARP + MAC + DDoS), DNS spoofing, and ultra-dense IoT deployments, and introduce a comprehensive hardware feasibility study and ablation analysis. Extensive testing across five real-world IoT datasets (CICIoMT2024, CICIoT2023, IoTID20, Edge-IIoTset, and TON_IoT) and twelve complex attack scenarios—including hybrid, adaptive, mimicry, and sequential attacks—demonstrates SFARP’s superior performance. On the CICIoMT2024 dataset, ADFDS achieved 98.3% accuracy, 97.6% precision, 98.9% recall, and a False Alarm Rate (FAR) of just 2.3%. On CICIoT2023, it maintained 96.0% accuracy and a 2.9% FAR, outperforming state-of-the-art models such as XGBoost and LightGBM across all key metrics. SFARP also demonstrated system-level advantages by reducing controller CPU usage by over 70%, minimizing packet loss by 90%, and maintaining end-to-end detection latency under 50 ms, even under high-volume attacks. Hardware evaluations on NetFPGA and Tofino ASIC confirm carrier-grade scalability, sustaining over 250 k concurrent flows with minimal memory overhead. By integrating programmable data-plane telemetry, adaptive ML-driven detection, and distributed mitigation, SFARP provides a scalable and hardware-feasible solution for real-time defense of SD-IoT infrastructures. It represents a practical step toward securing heterogeneous IoT deployments against evolving hybrid and multi-layer attacks.

## Introduction

The proliferation of the Internet of Things (IoT) has fundamentally changed the face of the technological landscape by incorporating billions of interconnected devices within the fabric of core industries such as healthcare, transportation, and manufacturing^1,2^. This new reality, while ushering in unforeseen levels of innovation and efficiency, has simultaneously fostered a vastly expanded attack surface, thus making these systems prone to severe security weaknesses^3^. Software-Defined Networking (SDN) has been hailed as the game-changing solution to address IoT’s massive scale and highly dynamic nature^4^. In abstracting the control plane concerning the data plane, SDN offers centralized network intelligence and programmability needed for orchestrating the vast number of devices and data commonplace in modern IoT applications^5^. However, the very design that infuses SDN-enabled IoT (SD-IoT) with dynamism is the same that introduces novel and complex security challenges, namely threats inherent with the nature and depth of multiple-layered attacks exploiting weaknesses within the network and the protocols^6^.

Among the most pervasive and damaging threats are Distributed Denial of Service (DDoS) attacks, which seek to disrupt network availability by overwhelming target resources with traffic^7,8^. Within SD-IoT environments, the impact of traditional flooding attacks is magnified, as attackers can leverage the vast number of often-insecure IoT devices to generate attack traffic at a catastrophic scale ^9^. The complexity of this threat is further compounded by the evolution of attack methodologies, which now include adaptive, coordinated, sequential, and varying-intensity patterns designed to evade static defenses^10^. A compelling and pernicious approach combines Address Resolution Protocol (ARP) spoofing with these flood attacks. A formidable force multiplier is ARP spoofing, a layer 2 attack in which a malicious entity forges ARP messages to illegitimately associate their MAC address with a genuine device IP address^11^. Successfully poisoning network device ARP caches allows an assailant to capture, alter, or, more importantly, reroute enormous traffic flows. In conjunction with a DDoS flood, an attacker can disable nearby network segments and extend the effect of an attack to create a sophisticated, hybrid menace moving across multiple networking layers that defies traditional models of protection^12–14^.

Traditional SDN architectural models often come up short in the face of these complex, multi-vector attacks. For instance, the most prevalent single-controller model presents a critical single point of failure. Its centralized design is simultaneously a performance bottleneck at scale and an ideal target for an attacker; bursting this single controller knocks out the entire network’s operating intelligence^15^. This weakness is seriously exacerbated by ARP spoofing, with which malicious traffic flows could be diverted strategically to cause maximum strain upon the central controller or significant network gateways. The inherent design weakness necessitates a shift from monolithic control planes to more robust and distributed ones.

These limitations reach beyond the architecture to the communication protocols themselves. Despite its foundational role in SDN, OpenFlow’s static, rule-based nature fundamentally limits it. It is not flexible enough to dynamically examine packet payloads or respond to the evasive, polymorphic behavior exhibited in modern hybrid threats that couple protocol-level manipulation, such as ARP spoofing, with volume-based flooding^16^. This inflexibility creates the need for a more programmable and intelligent data plane. The P4 programming language becomes an attractive answer to this shortcoming, providing unparalleled control over the packet-processing pipeline within the network switches. P4’s flexibility allows for the granular, stateful examination of traffic flows and ARP packets at line speed, and the resultant capability becomes the foundational capability for identifying and responding to threats without complete dependence upon the control plane^17^.

A new level of analytical capability is necessary to achieve the full potential of a programmable data plane. This is where machine learning (ML) is most valuable, providing a powerful tool for improving threat detection and mitigation. P4-programmable switches extract large-scale, high-frequency telemetry, which ML models use to identify subtle patterns and anomalous behavior indicative of malicious activity^10^. By combining the P4’s deep, packet-level programmability and the intelligent analytical capability of ML, it is possible to develop a defense system that can combat the increasing complexity of the increasingly subtle hybrid DDoS and ARP spoofing attacks. This integration is an essential shift in the SD-IoT security landscape, away from reactive, static defense and toward an intelligent, proactive, and multi-layered security position that can guarantee the resilience and reliability of future-generation IoT networks^18^.

### Problem domains and core research challenges

Detecting advanced DDoS and ARP spoofing attacks in SD-IoT networks presents significant research challenges that require innovative solutions. Major challenges are:

- **Dataset Limitations:** Existing benchmark datasets often lack the complexity of modern hybrid, adaptive, and sequential attack patterns, limiting their effectiveness for validating robust defense mechanisms.
- **Centralized Vulnerabilities:** Traditional single-controller SDN architectures introduce one point of failure, making them highly vulnerable to controller overload and network downing in a large-scale attack scenario.
- **Resource Constraints:** IoT devices and SDN controllers’ limited processing and memory capacity constrain the deployment of computationally intensive security tasks.
- **Protocol Inflexibility:** Inflexible protocols such as OpenFlow do not possess the dynamism needed for real-time threat response since they cannot be dynamically programmed for new or developing attack vectors at line rate.
- **Stealthy and Evolving Threats:** Attackers use more adaptive, dynamically varying-intensity, and low-rate methods that avoid fixed, threshold-based protection systems.
- **Scalability Requirements:** The exponential increase in IoT devices and traffic volume necessitates scalable security solutions that do not compromise performance.

### Research contributions and novelty

SFARP is designed to solve the above challenges using a multi-layered adaptive architecture. Its main contributions are.

- **Dynamic Flow Analysis Module (DFAM):** A P4-based module that executes efficient, real-time traffic and ARP analysis within the data plane, offloads the control plane, and facilitates rapid, low-latency feature extraction.
- **Adaptive Dynamic Flow Detection System (ADFDS):** A machine learning ensemble that utilizes the features from DFAM to accurately classify complex and adaptive attack patterns, integrating historical and live data to minimize false positives.
- **Distributed Adaptive Mitigation System (DAMS):** An integrated mitigant module that employs a multi-controller structure for handling threats in a distributed format, increasing response efficiency and general network resilience.
- **Programmability in the Data Plane using P4:** P4 produces bespoke, stateful rules for detection and prevention at line speed for both volumetric flooding and subtle ARP manipulations, going beyond conventional protocols’ inflexibility.
- **Thorough Experimental Validation:** The framework’s performance is validated across ten diverse attack scenarios, including hybrid and sequential threats, using modern IoT datasets to demonstrate its real-world efficacy and high detection accuracy.
- **A Scalable Multi-Controller (MCP) Architecture:** An inherently scalable design that addresses the vulnerabilities of centralized systems by distributing control logic, ensuring robust performance in large and complex SD-IoT environments.

## Literature review

Software-Defined Networking (SDN) increasingly controls smart devices as their use increases. SDN is more controllable and flexible, so it fits the Internet of Things (IoT) well. Nevertheless, this arrangement brings new security challenges as well. Two more significant problems are Distributed Denial of Service (DDoS) attacks and Address Resolution Protocol (ARP) spoofing. Both operate differently—DDoS floods a network with traffic to take it offline, while ARP spoofing tricks devices into sending packets where they should not be sent. Both can significantly affect the functionality and protection of IoT systems.

Various researchers have proposed machine learning (ML) and deep learning (DL) techniques that exploit SDN’s global network observation to combat DDoS attacks. For instance, in a work^19^, an adaptive ML model was developed that could effectively detect DDoS attacks. It performed well in test setups, but since it only used simulated data for testing, it may not behave similarly in practical situations. Another design, presented in^20^, exploited a deep learning model called FFCNN with SVM-based feature selection for detecting low-rate DDoS attacks. It performed well in accuracy but was restricted since it only exploited one set of datasets. Another similar investigation^21^. The study integrated a deep learning classifier with the OpenDayLight SDN controller, presenting satisfactory detection outcomes, although the evaluation datasets did not accurately reflect real IoT traffic.

Some papers, like^22^, incorporated hybrid methods for better detection, blending machine learning and entropy-based anomaly detection. Such methods identified odd-looking traffic well but only examined packets without realizing the entire picture. Some others, like^23^, investigated using edge-based deep learning to rapidly detect low-rate DDoS attacks with a lower delay and high accuracy.

While DDoS attacks are well-documented and well-researched, ARP spoofing is a significant problem too—just more discreet. Tricking the network into sending data intended for one device to another can result in stolen data or disruption of services. To counteract this, some researchers have explored lower-resource, simpler methods. In one piece of research^24^, a table-driven system was constructed to inspect ARP traffic with flow rules. The system detected attacks quickly and required minimal processing power, but it only addressed ARP spoofing. Another approach^25^ used Snort3 and TShark to detect ARP spoofing in real time, and it performed well for identifying attackers in a smart grid. However, it was not tested in larger, more diverse IoT environments.

Some researchers have tried to build systems that can handle DDoS and ARP spoofing. For instance, reference^11^ describes a complex architecture that combines machine learning, programmable switches (P4), and multiple SDN controllers. This system performed well on real IoT data and managed both attack types simultaneously. However, the setup was very complex and has not yet been tested on a larger scale. Other studies have focused on prevention. For example,^26^ introduced a system that uses static MAC-IP mapping to stop ARP spoofing before it happens. It is simple and effective, but doesn’t work well in dynamic environments where devices frequently join or leave. In^27^, decision trees were used with Wireshark to spot ARP spoofing based on live traffic features. It gave favorable results in controlled settings but might not keep up with fast-changing IoT networks.

Lastly, in^28^, an efficient ARP spoofing detection and mitigation approach was proposed to improve SDN security, effectively enhancing the network’s ability to identify and respond to such threats. However, the study lacks scalability evaluation and real-world deployment validation. There has been much progress in detecting DDoS and ARP spoofing in SDN-IoT systems. Most models work well in lab conditions and show high accuracy, but many still rely on outdated or artificial datasets. Also, only a few solutions aim to detect both types of attacks together. These gaps show that more flexible, scalable, and realistic security solutions are still needed—especially ones that can keep up with the changing nature of IoT traffic and threats. Table 1 compares current DDoS and ARP spoofing defense studies in SD-IoT, noting their strengths and weaknesses.

**Table 1:** Summary of Related Works on DDoS and ARP Spoofing Detection and Mitigation in SDN-IoT Environments.

Ref	Year	Approach	Key Strengths	Limitations
^19^	2022	Adaptive ML framework for SD-IoT DDoS detection	Achieves strong results in both detection and mitigation	Evaluation limited to artificially generated information
^20^	2022	FFCNN with SVM-based feature selection	Exhibits high efficiency for Low-Rate DDoS identification	A narrow focus on a single attack type limits its utility
^21^	2022	DALCNN using OpenDayLight SDN controller	Demonstrates strong capability in identifying DDoS threats	Data used poorly reflects real IoT network traffic
^22^	2022	Hybrid model with entropy and ML-based anomaly detection	Accurate and efficient in handling Distributed Denial-of-Service	Analysis restricted to examining packets in isolation
^24^	2022	D-ARP: Table-driven detection and response system for ARP spoofing	Precise and instantaneous detection with no chances of false positives or false negatives	Scope limited to ARP spoofing; lacks broader utility
^11^	2023	Hybrid ML with stateful P4 and distributed multi-controller SDN	Provides integrated detection and mitigation for DDoS and ARP using real-world IoT data	High architectural complexity and limited validation at large-scale deployments
^23^	2024	Edge-based hybrid DL detection	Performs effectively against attacks with low traffic rates	Effectiveness in diverse scenarios is not confirmed
^27^	2024	Decision tree-based ARP spoofing detection using Wireshark	High detection accuracy in real-time scenarios	Limited effectiveness in complex, adaptive topologies
^28^	2024	Improving Security in SDN through Efficient Detection and Mitigation of ARP Spoofing Attacks	Strengthening the network’s ability to detect and respond to threats accurately	Limited scalability analysis, lacking validation in the real world
^25^	2025	Snort3- and TShark-based NIDS for ARP spoofing in microgrids	Real-time attacker localization; protocol-aware detection	Limited to microgrid use; not tested in SD-IoT broadly
^26^	2025	Static MAC-IP mapping to prevent MitM-ARP attacks	Proactive spoofing prevention with minimal resource use	Limited dynamic adaptability in large IoT systems

### The core components of the SFARP framework

The following is a summary of the three fundamental modules of the intended security framework. Each module serves a distinct function in identifying and countering flooding DDoS attacks and ARP spoofing efforts in SD-IoT networks. Each module is designed to integrate with others, building a resilient, adaptive, and real-time protection solution using various SD-IoT environment layers, P4 programming, machine learning, and dynamic tuning methods.

### The system model

SFARP is an integrated three-module approach engineered to enhance the security, resilience, and performance of Software-Defined IoT (SD-IoT) networks. Each of the modules operates on various layers of a network architecture and comprises the following:

1. **Dynamic Flow Analysis Module (DFAM):** Executing directly within the P4-enabled switch data plane, DFAM offers intelligent, real-time traffic analysis optimized specifically for SD-IoT applications. DFAM primarily works to recognize and react to flooding attacks (such as TCP, UDP, ICMP, and HTTP floods) and ARP spoofing efforts. It utilizes a variable monitoring configuration developed using the Logically Connected State Table (LCST) design. Based on this design, LCST enables DFAM to aggregate and examine historical and real-time traffic details within fixed monitoring windows. Each of LCST’s state tables monitors predefined network statistics and ARP cache entries to detect discrepancies indicative of spoofing activity. DFAM dynamically optimizes its monitoring windows to remain responsive and quick depending on network activity. P4-enabled switches collect network statistics, temporarily store them, and examine them for anomalies. After a fixed-size monitoring window becomes full, DFAM takes relevant features from both traffic and ARP details and verifies them against predefined baselines. Adaptive Adjustment Mechanism (AAM) is triggered upon detecting suspicious activity or ARP spoofing. This mechanism adjusts the monitoring settings finely and updates P4 policies in real time to neutralize a threat before it can propagate.
2. **Adaptive Dynamic Flow Detection System (ADFDS):** Designed to operate mainly at the data plane, the Adaptive Dynamic Flow Detection System (ADFDS) is focused on detecting and mitigating both flooding DDoS attacks and ARP spoofing attacks in SD-IoT environments. ADFDS leverages real-time traffic and ARP monitoring, coupled with machine learning, to adaptively classify traffic patterns and to trigger dynamic mitigation responses. This module targets attack types such as HTTP, TCP SYN, UDP, and ICMP floods, in combination with ARP spoofing, using traffic and ARP features extracted from P4-enabled switches. The ADFDS workflow starts with initializing monitoring parameters, baselines, and ML models. The next phase includes capturing the traffic and ARP metrics saved in monitoring windows. This enables the system to detect various attack scenarios, including hybrid or adaptive attacks and ARP spoofing attempts. The ProcessWindowMetrics() function analyzes the data in the monitoring windows. The system will trigger the Adaptive Mitigation Strategy function if any ARP spoofing indicators or traffic anomalies are detected. The traffic and ARP features are combined and sent to the detection and mitigation modules, where a machine learning system classifies traffic and applies mitigation strategies.
3. **Distributed Adaptive Mitigation System (DAMS):** Across the data as well as the control plane, the Distributed Adaptive Mitigation System (DAMS) offers real-time as well as coordinated defenses against DDoS and ARP spoofing attacks within the SD-IoT network. DAMS utilizes the features of the SDN controllers with programmable P4 switches to allow for traffic, ARP monitoring, detection of attacks, and coordinated mitigation measures among all the impacted switches. DAMS begins by loading mitigation policies and establishing communications channels. If a threat has been identified, the DistributeAnomalies() function distributes the details to all the impacted switches. If the threat involves ARP spoofing, ARPMitigation() blocks the source. In any other case, the function TrafficMitigation() will implement policies to reduce the impact caused by the attacks. DAMS also utilizes a DynamicAdjustment() mechanism, which monitors mitigation effectiveness, adjusts policies accordingly, and updates all the impacted switches. In implementation, the P4-enabled switches enforce the traffic and ARP policies, with the controllers coordinating every action for optimized performance.

**Fig. 1:**
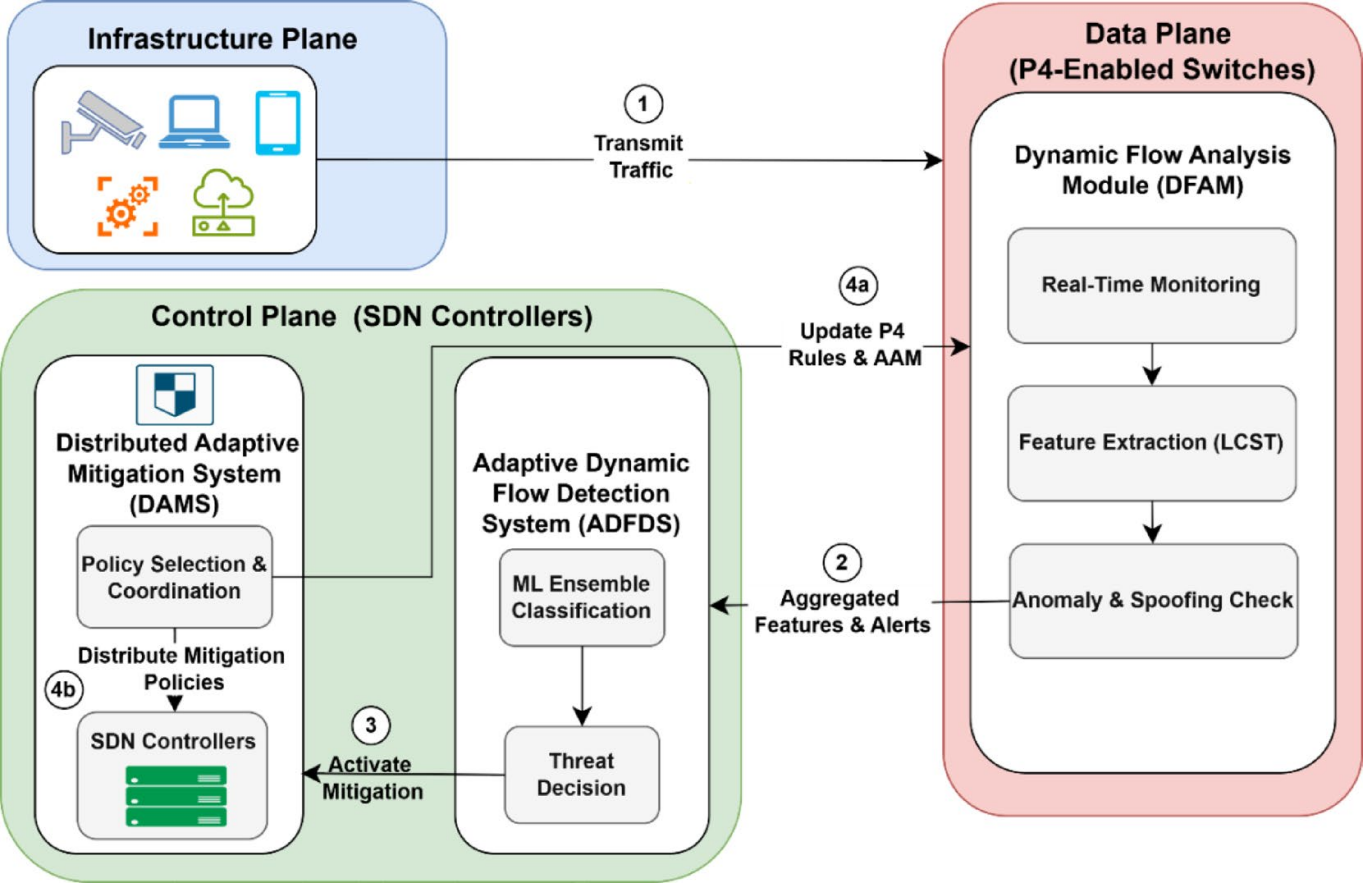
System Model of the Proposed SFARP Framework.

These three modules operate in a layered fashion, where traffic analysis, attack detection, and mitigation are performed in the data plane and coordinated using the control plane. The system’s distributed design guarantees a scalable, robust, low-overhead implementation fit for real-world SD-IoT environments. Figure 1 presents the layered architecture comprising the three main modules.

### The threat model

**Fig. 2:**
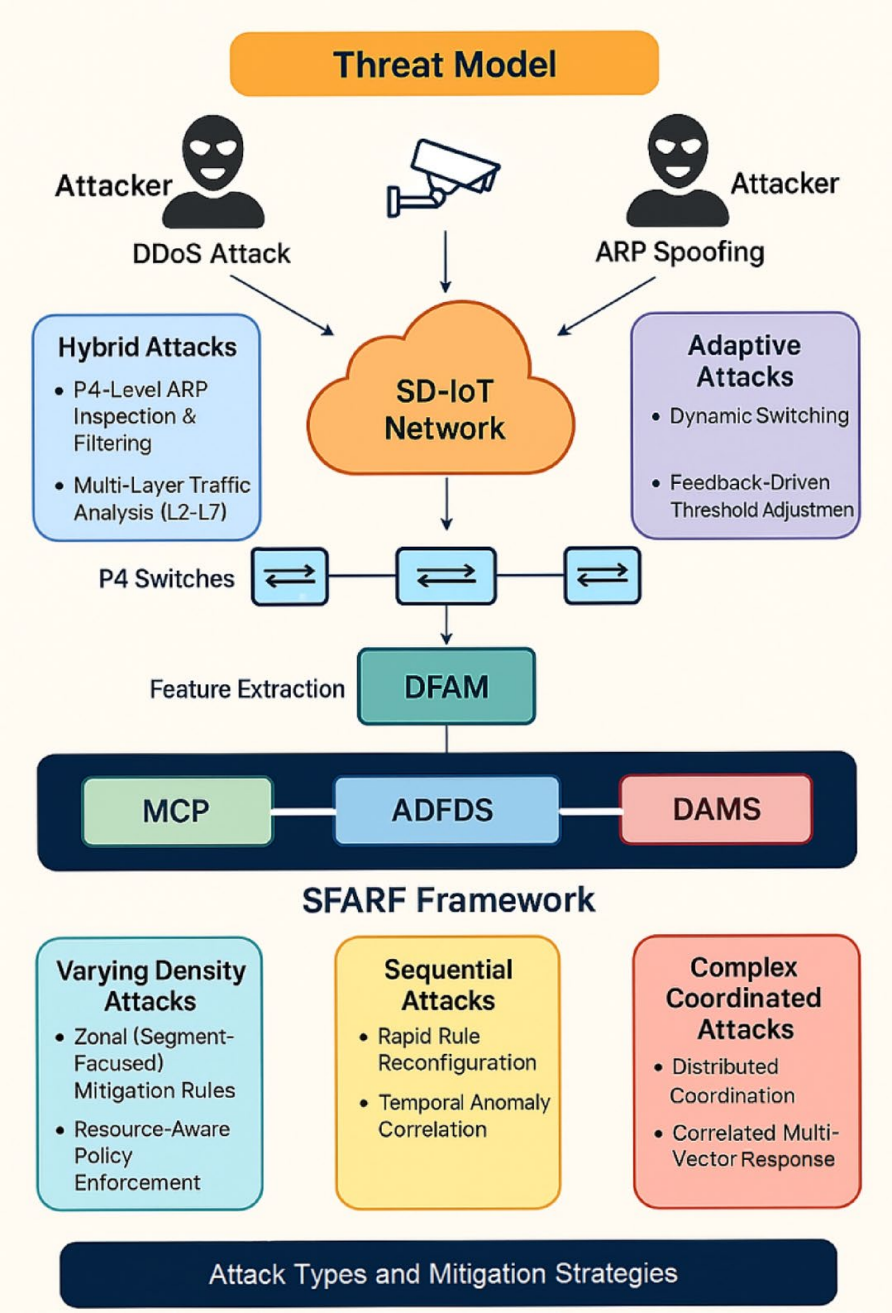
Threat Model and SFARP Mitigation Strategies.

The threat model considers coordinated attacks targeting IoT networks, combining ARP poisoning and flooding DDoS attacks to maximize impact and evade traditional defenses. These attacks can manifest in several forms: Hybrid attacks combine ARP poisoning to disrupt local network communication and redirect traffic with flooding attacks to overwhelm target devices or network segments. Adaptive attacks dynamically change their attack vectors, intensity, or targets in response to defensive measures. Varying density attacks target densely populated IoT segments, exploiting the increased vulnerability due to resource constraints and high traffic volume. Sequential attacks shift the attack focus from one target to another over time, making it harder to pinpoint the source and implement effective countermeasures. Finally, complex coordinated attacks combine multiple attack types (e.g., ARP poisoning, SYN floods, UDP floods) across different network segments, requiring sophisticated detection and mitigation strategies. These attacks exploit vulnerabilities at Layer 2 (ARP) and Layer 3/4 (network/transport), aiming to disrupt communication, degrade performance, and potentially gain control of IoT devices. The proposed threat model and SFARP mitigation strategies are illustrated in Fig. 2, which outlines the various attack types and corresponding defense layers in the framework.

### Dynamic flow analysis module (DFAM)

The Dynamic Flow Analysis Module, or DFAM, is an integral part of the intended secure management scheme, allowing for intelligent, real-time traffic analysis in SD-IoT networks. DFAM can identify and suppress adaptive multi-vector attacks, such as flooding attacks (TCP, UDP, ICMP, HTTP) and ARP spoofing attacks. With its dynamic and adaptive analysis features, the Logically Connected State Table (LCST) architectural design enables such detection. DFAM continuously adapts to variable traffic situations, maintaining high accuracy and efficiency in detecting malicious activity.

**Fig. 3:**
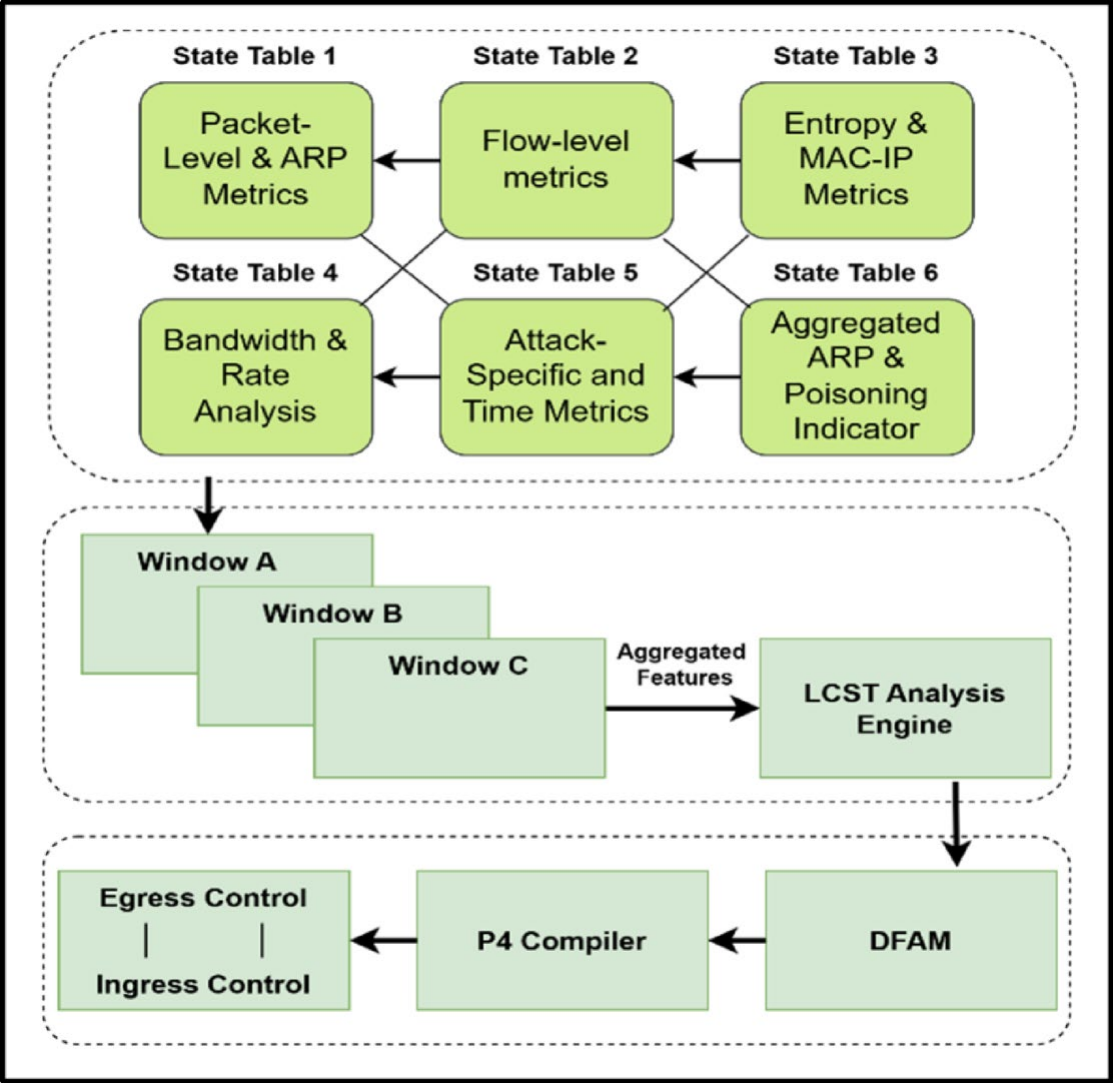
Implementation Scheme of Logically Connected State Table (LCST) in the DFAM Module.

The LCST structure is the central component in DFAM, consisting of interrelated state tables that store real-time and historical traffic characteristics within specified monitoring windows. As Fig. 3 indicates, every state table contains particular traffic measures that are continually updated and processed, including packet numbers, flow rates, and entropy values. The LCST further keeps track of ARP cache entries to identify inconsistencies that might signify ARP spoofing attacks. Through this interrelated composition, DFAM can retain an in-depth view of the network, making possible the revelation of subtle anomalies and overt attacks, including ARP poisoning .

DFAM’s Real-Time Adaptive Monitoring system breaks down traffic into non-intersecting monitoring windows adapted dynamically based on observed network activity. The monitoring windows serve data stores for history and real-time traffic information so that the system can identify, based on them, anisotropies indicative of threats. A radical packet rate increase across multiple flows might be indicative of initiating an intensive flooding attack, for example, and gradual, slow changes in the entropy values might be indicative of slow, progressive threats, and non-matched ARP cache entries might be indicative of an attempted ARP spoofing effort. DFAM’s Real-Time Analysis Engine acts upon this data to make precise and accurate discriminations between the normal fluxes of traffic and malicious activity, and now includes discriminations against ARP spoofing attacks.

At the core, the DFAM algorithm (**Algorithm 1**) sets up monitoring parameters using the SetupMonitoring() function. This sets baseline traffic patterns, the size of the windows, and the adjustment factors, besides initializing the monitoring for the ARP cache. These parameters provide the necessary flexibility for dealing with the bursts of traffic at high frequencies, slower attacks, and the new ARP attempts at poisoning. As it receives new traffic information, the system continuously adjusts these parameters to ensure DFAM remains prepared and always responds to the network’s needs.

As the traffic traverses the network, P4-enabled switches collect and save significant traffic details in the current monitoring period. We examine this information for anomalies such as flow direction, protocol type, and packet sizes. The system also keeps track of ARP traffic, with a special interest in mapping from the MAC address to the IP address. Such tracking is done by keeping track of modifications to ARP cache entries and searching for any incongruities, such as repeated MAC addresses with varying IP addresses or any change in the existing valid ones, both indicating attempted ARP spoofing.

When the monitoring window stands at its preset size, the WindowProcessing() function calculates significant traffic characteristics such as entropy, packet rate variance, and flow duration to determine anomalies. Furthermore, the WindowProcessing() function now looks for ARP traffic for evidence of spoofing. If ARP spoofing is detected, it immediately calls for the AAM. If not, it contrasts traffic characteristics with baseline thresholds and calls AAM if traditional anomalies are detected. For example, a significant drop in entropy would indicate coordinated efforts to launch an attack, unusual packet size distribution may indicate protocol-based exploit attempts, and non-conformity in ARP cache entries could indicate an ARP poisoning attempt.

Adaptive Adjustment Mechanism (AAM) combined with DFAM ensures that the monitoring windows and the parameters remain adaptive and agile for evolving traffic patterns and, now, for ARP spoofing detection. Upon any anomaly or ARP spoofing detection, the AAM dynamically changes the size of the windows, threshold values, and other monitoring parameters so the system remains agile and performs well. This is done using the Feedback Loop, which utilizes historical traffic data saved in the LCST for real-time monitoring strategy adjustment and learning the patterns of previously attempted ARP spoofing. The AAM now further includes the ability to quarantine the suspicious MAC addresses or IPs engaged in the spoofing activity.

DFAM’s programmability remains the foremost standing feature, made possible by the P4 Compiler and match-action rule programming. These rules govern how traffic is parsed, processed, and stored in the LCST, with precise feature extraction and real-time data handling. With the introduction of ARP monitoring, the P4-connected switches also receive and monitor ARP packets. Through P4’s programmability, DFAM dynamically takes on flow-specific and now ARP-specific monitoring policies and optimizes itself for any general and targeted attack detection.

After detecting an anomaly or ARP spoofing, the aggregated traffic features and ARP spoofing flags are exported to the framework’s detection and mitigation module using the FeatureAggregationAndExport() function. This function creates an integrated dataset for subsequent use by machine learning models. These models classify the traffic as malicious or benign, initiate quarantine measures upon detecting ARP attacks, and furnish network administrators with actionable recommendations.

**Figure:**
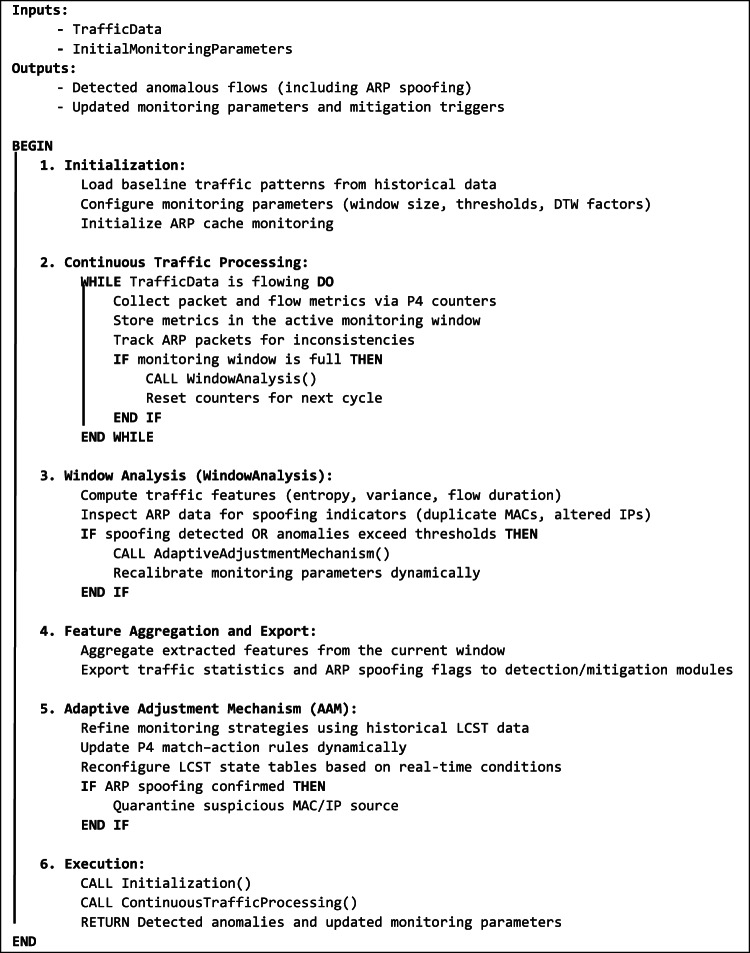
Algorithm 1: Dynamic Flow Analysis Module (DFAM) Workflow.

The Logically Connected State Table (LCST) provides context-aware analysis so that DFAM can detect patterns in multiple flow and time intervals. The LCST maintains a synchronized view of the associated traffic metrics and ARP cache data, allowing DFAM to detect complex, multi-vector attacks and ARP-based attacks accurately. For example, a TCP SYN packet and an ICMP traffic surge might indicate a coordinated flooding attack. In contrast, an ARP reply packet surge with non-matching source MAC addresses might be an ongoing ARP spoofing attack. The LCST structure allows DFAM to track and analyze these patterns adequately. DFAM provides a robust, adaptive, and intelligent mechanism for real-time traffic monitoring and threat detection in SD-IoT networks. The integration of LCST, adaptive monitoring, P4 programmability, and ARP spoofing detection and response ensures that DFAM remains a cutting-edge solution for mitigating complex attack scenarios and safeguarding network stability and security.

Table 2 outlines a robust feature set crucial for real-time detection and mitigation of complex network attacks, encompassing advanced DDoS flooding and ARP spoofing scenarios. For completeness, the full version of Table 2, including detailed feature descriptions, storage and processing requirements, and extended attack mappings, is provided in the supplementary materials. This includes sequential, coordinated, hybrid, adaptive, and varying-intensity attacks. Each feature is carefully selected to capture specific attack behaviors, patterns, and anomalies across multiple protocols (TCP, UDP, HTTP, ICMP) and malicious ARP activities. The feature set enables a granular network traffic analysis, facilitating the identification of subtle and overt deviations from normative patterns. Core metrics quantifying traffic volume, such as Packets Per Second (PPS), Bytes Per Second (BPS), and Flows Per Second (FPS), are essential for detecting high-velocity, large-scale flooding events and any amplification resulting from ARP redirection. Concurrently, features that assess traffic dynamics, including Inter-Packet Delay (IPD) and Sliding Window Rate (SWR), are critical for identifying variations in attack intensity and capturing adaptive behaviors. Furthermore, statistical measures of randomness, such as Entropy of Packet Size (ETP) and Traffic Randomness (TR), serve to uncover the sophisticated evasion tactics employed by attackers who manipulate packet uniformity and distribution.

**Table 2:** Core P4-Extracted Features for Detecting and Mitigating Hybrid Attacks.

Index	Feature	Description	Granularity	Relevance to Attacks
1	PL–Packet Length	Size of packets traversing the network	Packet-level	All floods; MITM variations
2	IPD–Inter-Packet Delay	Time gap between consecutive packets in a flow	Packet-level	Slow/variable floods, hybrid attacks
3	PPF–Packets Per Flow	Number of packets per flow in a time window	Flow-level	Coordinated/hybrid floods
4	BPS–Bytes Per Second	Total bytes transmitted per second	Flow-level	Bandwidth-intensive floods, adaptive attacks
5	FPS–Flows Per Second	Number of unique flows observed per time window	Flow-level	High-intensity coordinated/hybrid floods
6	ETP–Entropy of Packet Size	Randomness of packet size distributions	Packet-level	Adaptive/coordinated floods
7	BTU–Bandwidth Utilization	Percentage of network bandwidth consumed	Flow-level	High-utilization sequential and hybrid floods
8	RST–Reset Packets Ratio	Ratio of TCP RST packets within flows	Packet-level	SYN floods, hybrid adaptive floods
9	ACK–ACK Packets Ratio	Proportion of TCP ACK packets	Packet-level	TCP floods, coordinated/hybrid scenarios
10	PPS–Packets Per Second	Overall packet rate in a given interval	Flow-level	High-speed floods, hybrid, adaptive
11	LTP–Large Traffic Packets Ratio	Proportion of oversized packets	Packet-level	UDP/HTTP floods; congestion attacks
12	OWD–One-Way Delay	End-to-end delay between source and destination	Packet-level	Timing manipulation; sequential floods
13	FR–Flow Repetition Rate	Repetitive flow patterns within a window	Flow-level	Coordinated/hybrid/adaptive floods
14	SWR–Sliding Window Rate	Packet/flow intensity over sliding windows	Window-level	Sequential/adaptive attacks
15	TR–Traffic Randomness	Statistical randomness in flows/packets	Packet-level	Adaptive and stealth floods
16	UFP–Unique Flow Patterns	Distinct flow identifiers (SrcIP, DstIP, Ports)	Flow-level	Coordinated/hybrid floods
17	NTI–New Traffic Indicators	Spikes in new/unrecognized flows	Flow-level	Sequential, hybrid adaptive floods
18	TTF–Total Traffic Features	Aggregated traffic metrics in a window	Window-level	Complex coordinated scenarios
19	ATP–Attack-Specific Patterns	Flood signatures (SYN, HTTP, ICMP)	Flow-level	All attack types and intensities
20	AMC–ARP MAC Changes	Number of ARP entry changes in a window	Network-level	ARP spoofing, hybrid adaptive ARP attacks
21	APC–ARP Packet Count	Abnormal ARP request/response spikes	Network-level	Sequential/hybrid ARP floods
22	DMP–Duplicate MAC-IP Pairings	Duplicate MAC with different IPs	Network-level	All ARP spoofing scenarios
23	API–ARP Poisoning Indicator	Computed poisoning score using AMC, APC, DMP	Network-level	All ARP-based adaptive/hybrid attacks

The amount of granularity for a given feature—packet, flow, or window level—is explicitly specified so that each would be readily implementable in real-time, dynamic settings. Types of attack indicators, such as reset packet ratios (RST) and unique flow patterns (UFP), permit the precise identification of well-known attack footprints, such as SYN floods and coordinated bursts of traffic. The table further describes important features explicitly designed for ARP spoofing detection, like the ARP MAC Changes (AMC), ARP Packet Count (APC), the Duplicate MAC-IP Pairings (DMP), and the composite ARP Poisoning Indicator (API). Incorporating these ARP-focused metrics with the standard traffic analysis presents an inclusive, multi-layered threat landscape perspective. It allows for the increased versatility and effectiveness of the detection model within complicated situations. Importantly, the table further considers each feature’s storage and processing dimensions to reach an equilibrium between detection precision and performance overhead, which is essential for resource-limited SD-IoT networks.

**Fig. 4:**
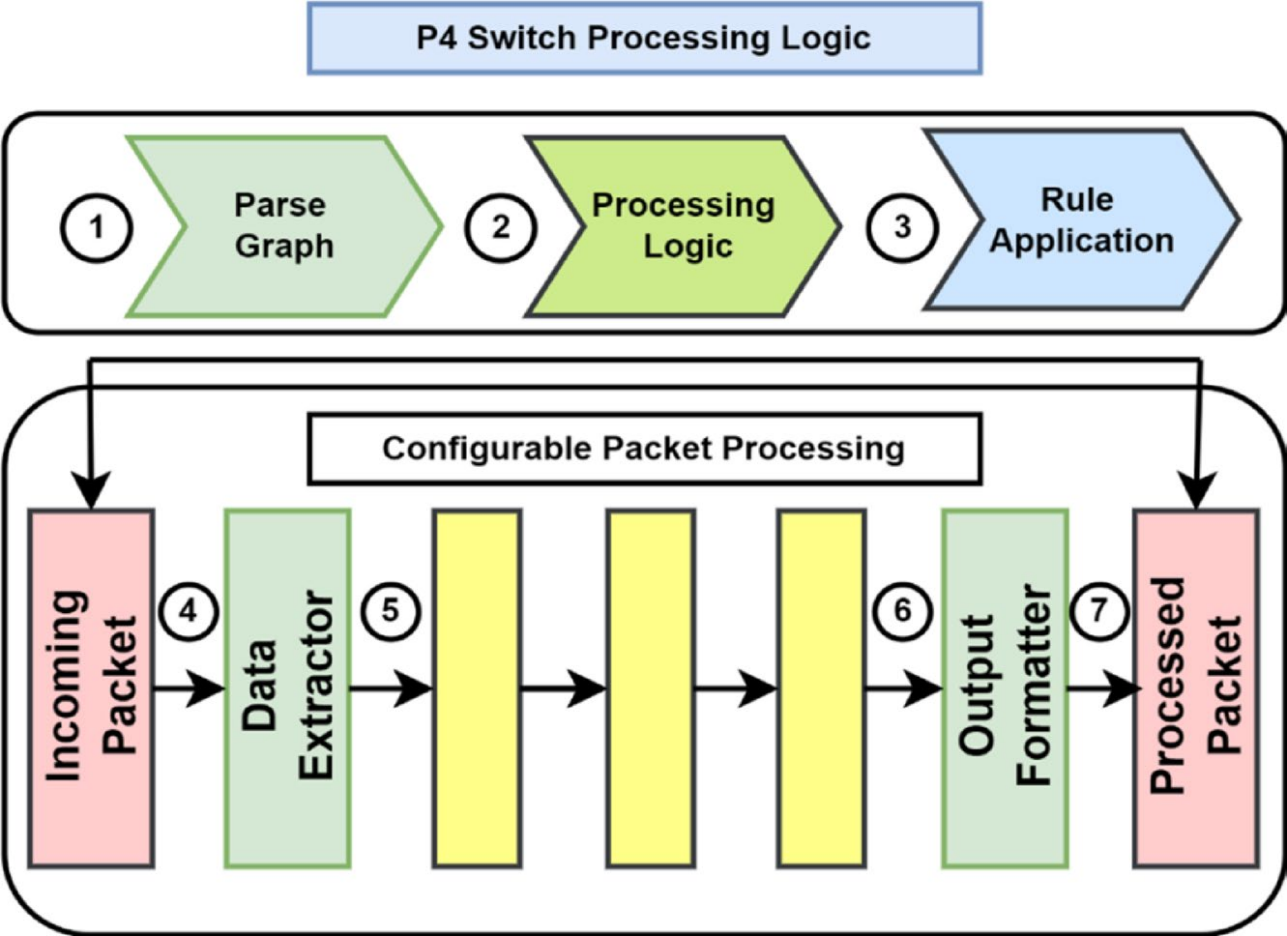
Architectural Model of the P4 Programmable Processing Pipeline.

**Fig. 5:**
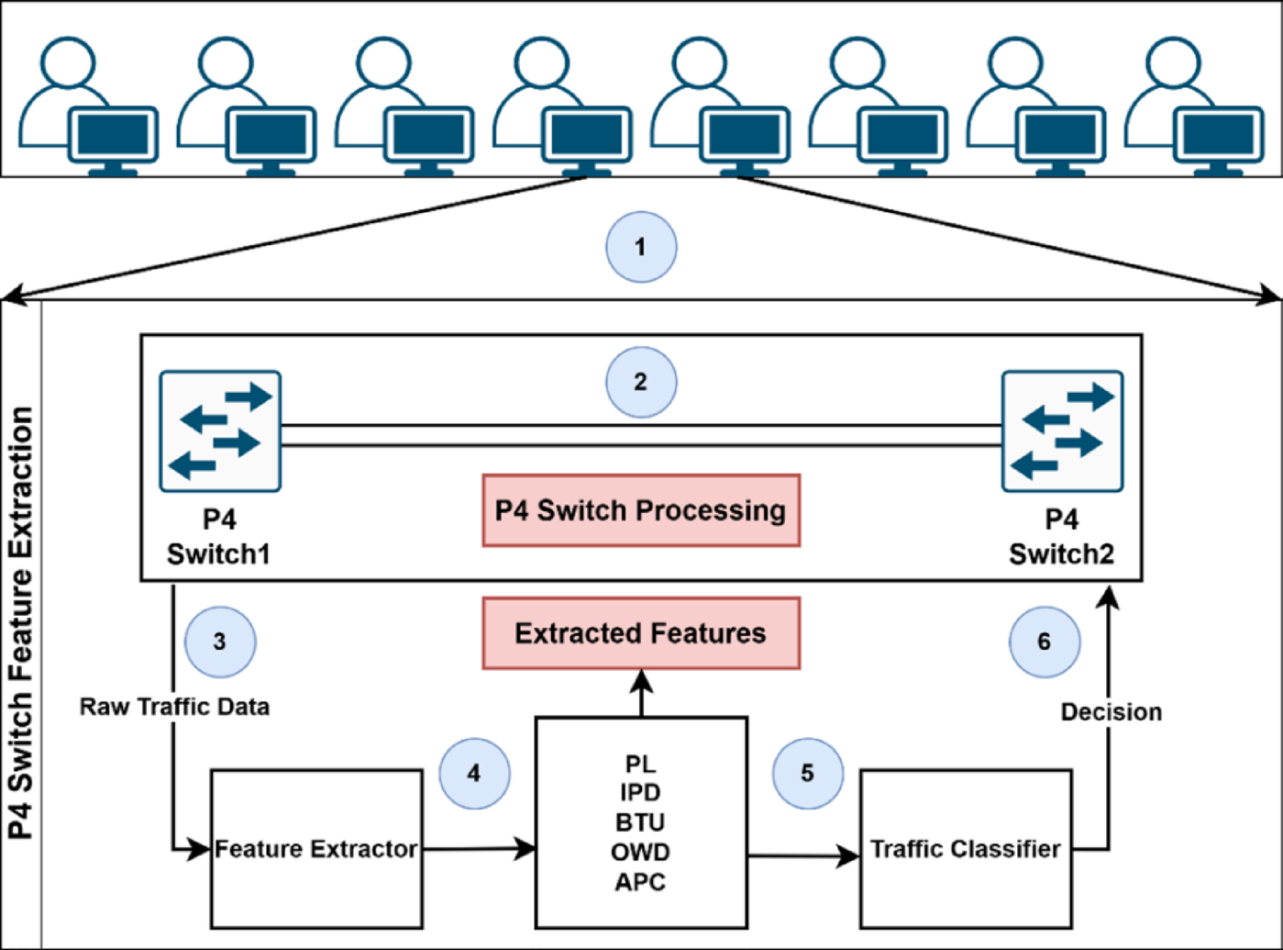
P4-Based Feature Extraction and Classification Mechanism.

P4 provides a highly programmable architecture for direct feature extraction within the data plane. P4 programs run through ingress and egress pipelines, enabling real-time packet inspection and traffic analysis. The ingress pipeline uses match-action tables to gather features like packet rates, flow durations, and entropy. Dynamic state tables maintain aggregated metrics within adaptive monitoring windows, detecting fluctuations in traffic patterns, which indicate DDoS attacks. This method reduces dependence on the SDN controller. The egress pipeline complements this approach by monitoring outgoing traffic and managing suspicious traffic in real time. Handling ingress and egress traffic concurrently minimizes overhead on the SDN controller, and the system responds faster to evolving attacks. Figure 4 presents the P4 program pipeline’s architecture, which is the foundation for the framework’s feature extraction and classification workflow, illustrated in Fig. 5.

### The adaptive dynamic flow detection system (ADFDS) module

The Adaptive Dynamic Flow Detection System (ADFDS) is designed to address the complex challenge of detecting and mitigating both flooding DDoS attacks and ARP spoofing attacks in SD-IoT environments. ADFDS combines real-time traffic monitoring, which is enhanced by tracking the ARP cache, with advanced machine learning to classify traffic patterns and implement dynamic mitigation measures adaptively. ADFDS focuses on key attack types, including HTTP, TCP SYN, UDP, ICMP floods, and ARP spoofing, ensuring a comprehensive approach to handling diverse and evolving attack scenarios, including complex hybrid attacks. An effective anomaly detection and response regime is provided by utilizing traffic and ARP features abstracted from P4-programmable switches and combining them with dynamic optimization procedures.

InitializeMonitoring() starts the ADFDS workflow by setting up monitoring parameters, machine learning models, and an initial ARP cache baseline. In the subsequent step (*AnalyzeTraffic()*), incoming traffic is analyzed. P4-programmed switches observe traffic, and ARP statistics concerning MAC-IP bindings are tracked. Such statistics are stored in tracking windows to reflect temporal traffic behavior. These windows allow the system to identify dynamically evolving attack scenarios, such as hybrid or adaptive flood attacks and ARP spoofing efforts, as a function of traffic and ARP statistics evolution over time. Features such as packet rates, flow entropy, inter-arrival times, ARP MAC address changes, duplicate MAC-IP pairings, and ARP packet counts are key to identifying anomalies and ARP poisoning attempts. The system utilizes *ProcessWindowMetrics()* to analyze the data in the monitoring window. First, the system evaluates any ARP spoofing indicators, and if that is the case, the system will trigger the Adaptive Mitigation Strategy. Otherwise, the system derives traffic feature metrics and compares these derived traffic metrics against baseline thresholds. If anomalies are detected, the Adaptive Mitigation Strategy will be triggered (*AdaptiveMitigationStrategy()*). The system will proceed with the next cycle if neither traffic anomalies nor ARP spoofing attacks are detected. The system leverages FeatureAggregation(), aggregating traffic and ARP features extracted from the current monitoring window, sending the aggregated data to the detection and mitigation modules.

The algorithm (**Algorithm 2**) has a machine-learning-based detection function named Phase 6: Machine Learning Based Detection. At the heart of ADFDS is the ensemble-based detection procedure that utilizes an array of base classifiers, including Random Forest (RF), Support Vector Machine (SVM), Extreme Gradient Boosting (XGB), Gaussian Naive Bayes (GNB), and k-Nearest Neighbors (kNN). The base classifiers are trained with the optimized attribute sets, and the initial weight is evenly distributed. Each base classifier produces predictions for new incoming traffic and computes the confidence score for the prediction based on the accuracy it achieved at the time of validation. The meta-optimizer, designed as a Dynamic Neural Network (DNN), pools these predictions and dynamically tunes the weightage for each base classifier based on their instantaneous performance. During real-time detection, ADFDS processes the traffic streams as P4-enabled switches and monitors ARP-related information. The algorithm dynamically derives features and utilizes the ensemble for tagging packets as ‘Benign’ or ‘Attack.’ The meta-optimizer corrects predictions by favoring those with a higher confidence score, ensuring that packets are accurately classified during intense attacks. A weightage-based aggregation formula decides the final output so that ADFDS can adaptively shift the focus whenever the patterns of the attack change and adapt for ARP spoofing when it is part of the system.

This algorithm uses a decision logic system to compare the combined confidence value with a predetermined threshold. Suppose the value is greater than the threshold. In that case, the traffic is flagged as malicious, and the system initiates the mitigation action with AdaptiveMitigationStrategy (AMS), such as rate-limiting, redirecting flows, or blocking the origin of ARP spoofing, minimizing the impact on positive traffic. However, if the value dips below the threshold, the traffic is marked harmless and forwarded without disruption. This adaptive decision-making process balances security with operational efficiency. The AMS was also strengthened with mitigation for attacks launched with ARP through source MAC address/IPv4 address quarantine, P4 rule revocation for spoofed traffic, and adjustment in the LCST for new conditions.

ADFDS incorporates a feedback loop to sustain its efficacy over time. In case performance deterioration is identified, the system adjusts the weights for the classifier, updates the meta-optimizer, and adjusts the detection thresholds to align with realistic traffic patterns. Such adaptability is critical for adapting to dynamically changing attack tactics and attaining high detection rates in adaptive network scenarios. The performance measurement phase sums up the performance of ADFDS in terms of various measures, such as accuracy and detection latency. Such measures identify the algorithm’s competence for detecting, finding, and preventing flooding attacks and ARP spoofing scenarios while minimizing the impact on normal network operations. The ADFDS is an effective solution for protecting SD IoT networks from highly advanced DDoS attacks and ARP-based attacks. It uses the learning framework for ensembling, dynamical optimization, and online learning. Its modularity guarantees robustness and scalability, making it suitable for implementation in heterogeneous IoT infrastructures.

The datasets employed for this research contain detailed network traffic features, each instance labeled with its corresponding class (either standard or a specific attack type). The CICIoT2023 dataset, used here as a representative example and detailed in Table 3, presents a wide array of attack categories characterized by a significant class imbalance. This disparity in the number of instances per class poses a considerable challenge for training effective machine learning models. Class imbalance can introduce a strong bias in the model, causing it to favor the over-represented majority classes while struggling to identify the under-represented minority ones accurately. Such a bias severely limits a model’s capacity to generalize to real-world network environments where the distribution of threats is inherently unpredictable.

**Table 3:** Class Distribution of the CICIoT2023 Dataset After Resampling for Model Training.

Attack Category	Total Instances	Original Training Instances	Adjusted Training Instances	Testing Instances	Resampling Strategy
HTTP flood	27,510	19,257	40,000 (+ 108%)	8,253	Over-sampling
UDP flood	5,410,500	4,328,400	4,328,400 (0%)	1,082,100	No change
RSTFIN flood	4,521,300	3,617,040	3,617,040 (0%)	904,260	No change
PSHACK flood	4,015,800	3,212,640	3,212,640 (0%)	803,160	No change
TCP flood	4,389,100	3,511,280	3,511,280 (0%)	877,820	No change
ICMP flood	7,195,200	5,756,160	150,000 (-97%)	1,439,040	Under-sampling
UDP Fragment	275,350	220,280	220,280 (0%)	55,070	No change
ACK Fragment	281,900	225,520	170,000 (-25%)	56,380	Under-sampling
SYN flood	4,033,400	3,226,720	250,000 (-92%)	806,680	Under-sampling
SlowLoris	22,150	17,720	35,000 (+ 98%)	4,430	Over-sampling
ARP spoofing	305,400	244,320	150,000 (-39%)	61,080	Under-sampling
DNS spoofing	177,800	142,240	70,000 (-51%)	35,560	Under-sampling

To counteract this issue, we implemented a strategic resampling methodology that involved oversampling for the underrepresented and overrepresented attack classes, thereby ensuring a more balanced and effective learning process for our model. In our adjusted training dataset, we strategically increased the number of instances for minority classes, such as HTTP flood and SlowLoris, which were initially present in very low quantities. On the contrary, we used random undersampling to limit the effect of overwhelmingly prevalent classes, such as the ICMP and SYN floods, which might otherwise dominate the learning stage. This method was carefully crafted to balance forming a representative learning set and maintaining the natural properties of the data.

This resampling approach successfully decreased the Class Imbalance Ratio of the training data and created a more balanced learning environment for our ensemble detection model. Class balancing in this way significantly improves the model’s capability to learn about the unique features of various types of threats, from high-volume floods to low-key spoofing attacks. This, in turn, advances its generalizability to new, unseen data with varying class distributions. We must take a moment to point out that our test set was left in its natural, unchanged configuration for an unbiased and thorough assessment of the model’s performance on realistic data. We explain our final distribution of balanced attack categories and our chosen resampling approach in Table 3**.** This table indicates the total instances for each attack, original and balanced-out training instances with a corresponding percentage change, and the resampling approach used. These resampling adjustments are integral to making the model more generalizable, as they allow for learning from all attack types without favoring or neglecting an individual category.

**Figure:**
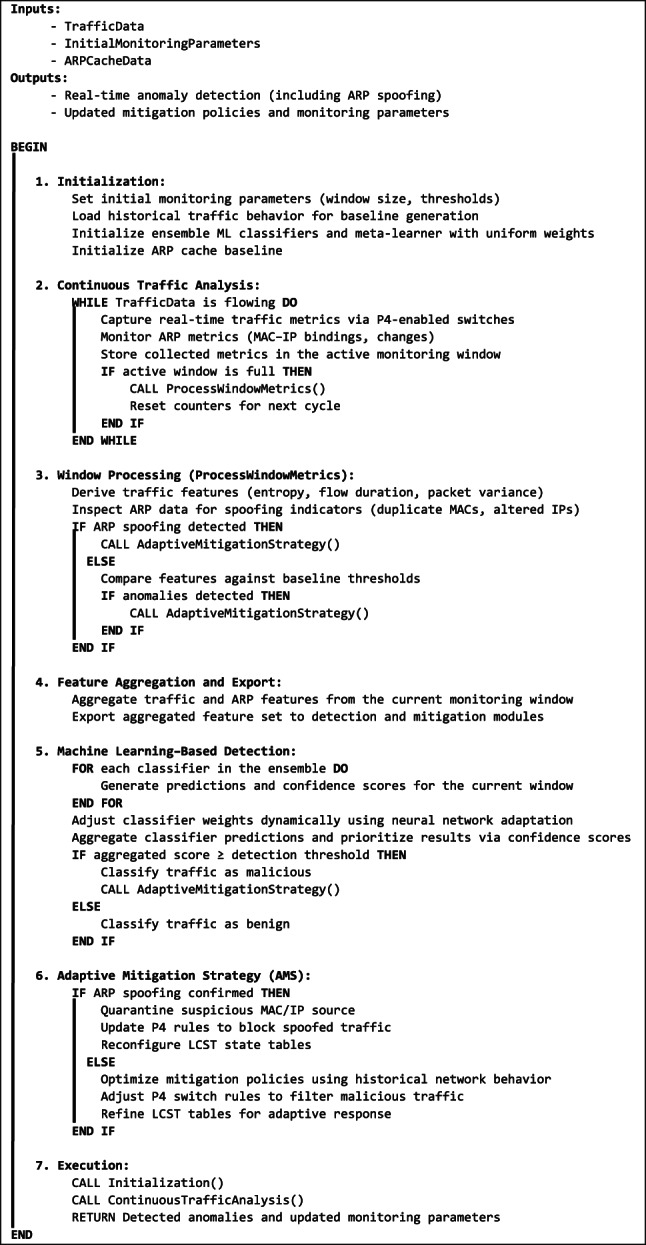
Algorithm 2: Adaptive Dynamic Flow Detection System (ADFDS) Workflow.

### Distributed adaptive mitigation system (DAMS) module

The DAMS algorithm offers real-time, cooperative, and dynamic protection against ARP spoofing efforts and flooding DDoS attacks for SD-IoT networks. DAMS leverages SDN controller distributed functions and programmable P4-based switches to accommodate real-time ARP, traffic monitoring, adaptive anomaly detection, and coordinated mitigation. DAMS addresses a broad class of attacks, such as TCP, UDP, ICMP, HTTP, SYN floods, and ARP spoofing attacks, with intelligent traffic analysis and flow management on demand.

**Figure:**
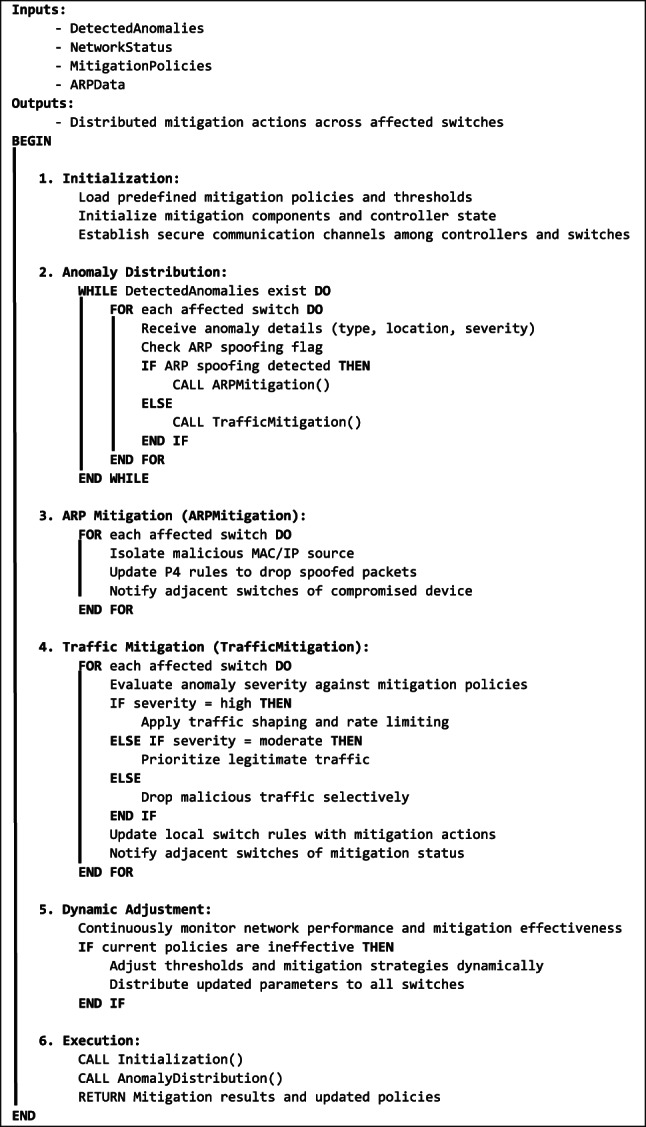
Algorithm 3: Distributed Adaptive Mitigation System (DAMS) Workflow.

The algorithm (**Algorithm 3**) begins with InitializeDAMS(), which loads mitigation policies and thresholds, sets up communication channels, and prepares mitigation components. During attack detection, the DistributeAnomalies() function receives anomalies and identifies the affected switches. If the anomalies are related to an ARP spoofing attack, then ARPMitigation() is invoked; otherwise, TrafficMitigation() is called. The ARPMitigation() function isolates the source of the attack, updates the P4 switch rules, and notifies adjacent switches of the malicious activity. The TrafficMitigation() function evaluates the attack severity and applies policies to suppress the malicious traffic, either by traffic shaping, rate limiting, prioritization, or dropping suspicious packets. All affected switches have been updated with mitigation actions.

DAMS also features DynamicAdjustment(), which monitors the effectiveness of the implemented mitigation strategies. If the system detects ineffective mitigation policies, it adjusts the thresholds and policies, updating all the switches with the new parameters. The system operates at both the control and data plane levels. At the control plane level, SDN controllers coordinate and share mitigation logs, continuously adapting and improving the system’s mitigation strategies. The P4-enabled switches execute the controller-provided policies on the data plane, dropping suspicious packets at line speed. The distributed nature of DAMS ensures the system remains scalable and resilient to multi-vector attacks.

This adaptive and distributed approach ensures that DAMS is effective against prolonged or evolving attacks and can maintain optimal network performance. DAMS provides a comprehensive solution by combining real-time monitoring, adaptive mitigation, and collaborative defense strategies, guaranteeing high performance in handling complex attack scenarios.

## Experimental setup and performance evaluation analysis

### Experimental setup

To evaluate the effectiveness of the proposed security framework, we implemented a multi-layered SD-IoT network topology using Mininet-WiFi, as depicted in the network diagram. This setup simulates a realistic environment and encompasses several key components. The control plane is structured hierarchically, consisting of a central controller, a backup controller for redundancy, and two domain controllers that each manage distinct network areas. The data plane mirrors this hierarchy with four programmable P4-enabled switches (SW1, SW2, SW3, and SW4) managed by their corresponding domain controller. This two-tiered system for control and data ensures a realistic implementation of SDN concepts.

The Central Controller communicates directly with both Domain Controllers and the Backup Controller; the Backup Controller also communicates with both Domain Controllers. This interconnectedness provides a central point for policy configuration and a fallback system if one of the controllers goes down. Each domain controller directly manages two P4-enabled switches, with Domain Controller 1 overseeing SW1 and SW2 and Domain Controller 2 overseeing SW3 and SW4. These switches are directly connected to access points 1, 2, 3, and 4, which provide wireless connectivity to the IoT devices. Twenty host devices, labeled H1 through H20, are distributed across four distinct IoT domains, each with five devices managed by its corresponding access point. These domains are associated with the P4 switch nearest to the network (SW1 is connected to IoT Domain 1, SW2 is connected to IoT Domain 2, and so on) and also have domain descriptions for clarity (IoT Domain 1, IoT Domain 2, etc.), and the P4 switches are associated with their two nearest IoT domains as well (SW1 is connected to IoT Domain 1 and IoT Domain 6, and so on).

This complete setup was created using Mininet-WiFi, a powerful open-source network emulator that supports both wired and wireless components and allows us to emulate P4 switches realistically, which is critical for the evaluation. For traffic capture, Wireshark was integrated into the Mininet-WiFi environment, enabling real-time data flow analysis between devices in the network. The hping3 command-line tool was used directly within the simulated devices to generate legitimate traffic and simulated DDoS attack scenarios, enabling various flooding attacks, such as HTTP, TCP SYN, UDP, and ICMP floods with different intensity levels.

**Fig. 6:**
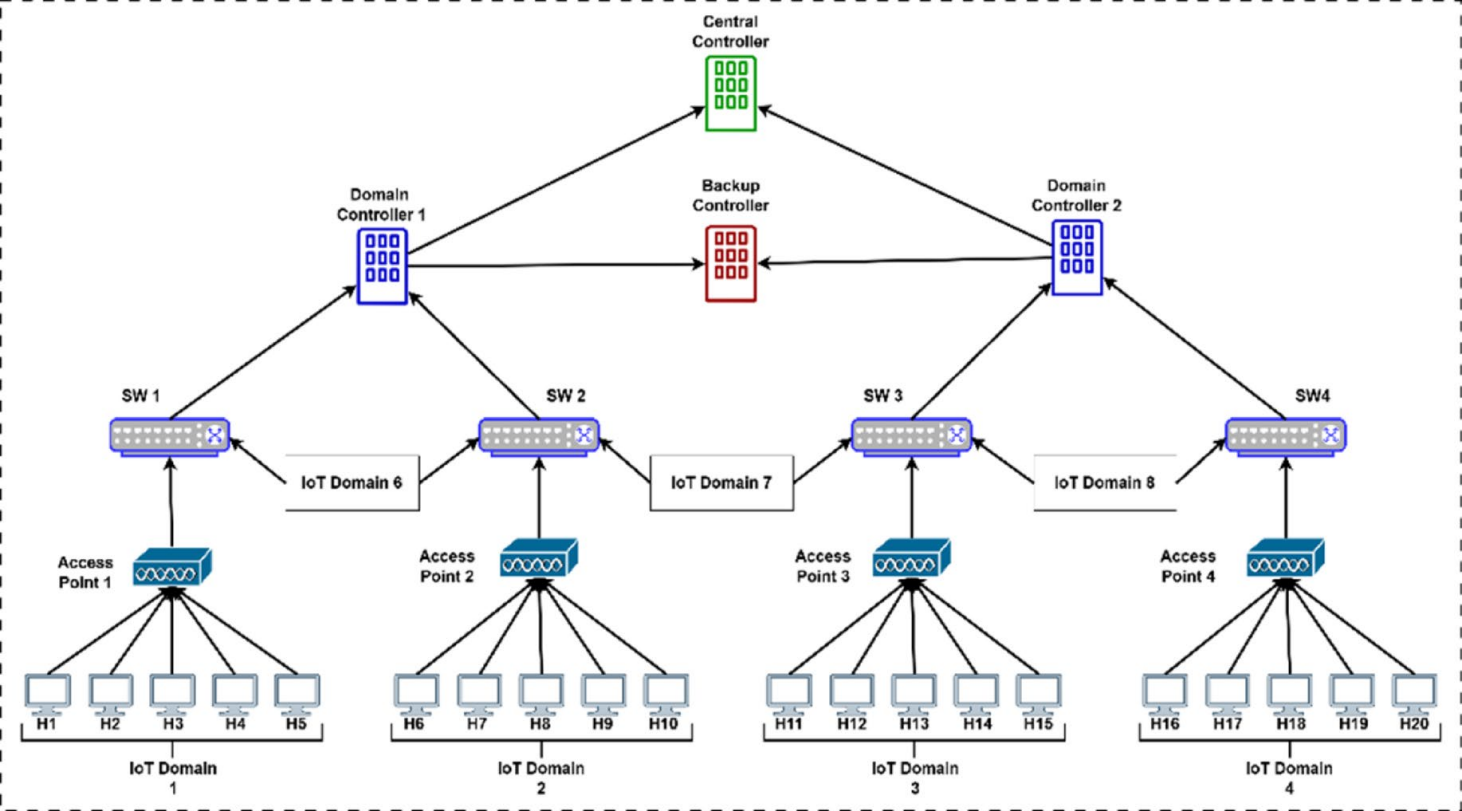
The Hierarchical and Scalable Test Topology for the SFARP Defense Framework.

All the experiments were conducted on a machine using an Intel Core i7 processor, 16 GB of RAM, and an SSD hard drive. Our findings show that the experiment can be executed effectively on any machine with similar specs, such as an Intel Core i5/i7 processor, 8 GB to 16 GB of RAM, and an SSD/NVME hard drive. This experimental environment provides a robust and realistic platform for credibly evaluating the proposed security framework. This approach seamlessly integrates all essential components to yield easily reproducible results. Figure 6 illustrates the hierarchical and scalable test topology for the SFARP defense framework.

### Experimental test scenarios

**Fig. 7:**
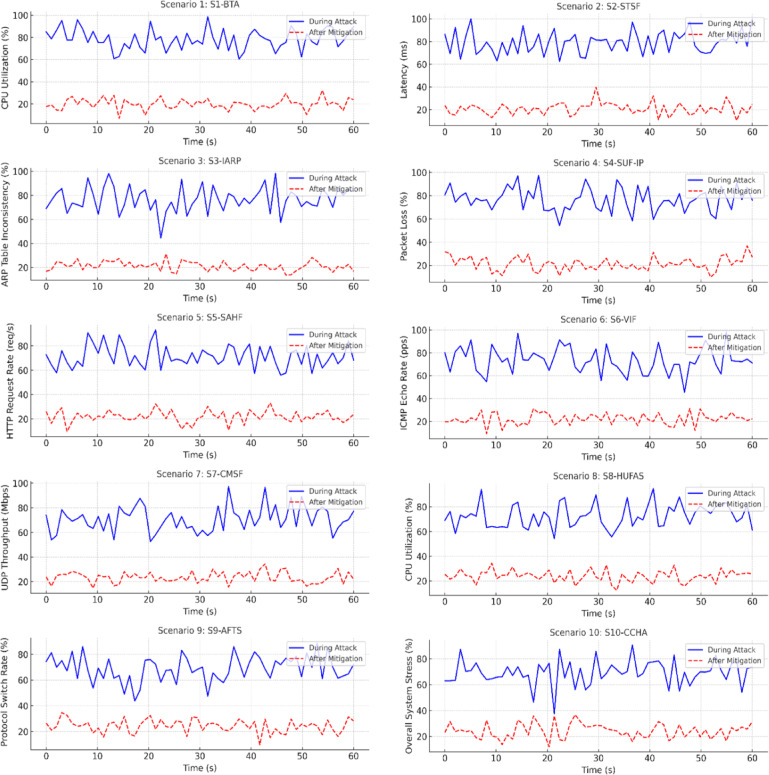
Impact of SFARP’s Mitigation on Key Performance Metrics Under Various Attack Scenarios.

This section details ten test scenarios designed to rigorously assess the ADFDS system’s capabilities in detecting and mitigating a wide range of flooding and ARP-based attacks. These scenarios, illustrated in Fig. 7, span benign baseline conditions to adaptive, coordinated, and hybrid threats. Each includes a description of the attack mechanics, network impact, and the system’s detection and mitigation behavior.

**Scenario 1: Baseline Traffic Analysis (BTA).** This scenario reflects a clean and stable network environment with no active threats. Traffic normally flows between devices, with consistent MAC-IP relationships and expected communication patterns. While the detection engine remains inactive, the traffic analysis module observes standard behavior, confirming the absence of false positives. The mitigation module remains in monitoring mode, ensuring that legitimate traffic is not disrupted. This scenario establishes the system’s ability to operate silently and accurately under normal network conditions.

**Scenario 2: Single Host TCP SYN Flood (STSF).** A classic TCP SYN flood is launched from a single compromised device targeting one IoT node. The attacker overwhelms the target with half-open connections, attempting to exhaust its resources. The traffic analysis module detects an unusual surge in SYN packets, while completed handshakes remain low. The detection module recognizes this imbalance and classifies the pattern as a flood. The mitigation module activates rate-limiting rules at the network edge, reducing the attack’s intensity and protecting the targeted device without affecting other flows.

**Scenario 3: Isolated ARP Spoofing Attack (IARP).** In this scenario, the attacker attempts to compromise the network solely through ARP spoofing, without accompanying flooding activity. Falsified ARP replies are injected into the network to mislead devices and reroute traffic. The traffic analysis module notices inconsistencies in MAC-IP bindings, especially those that change too frequently or contradict the ARP cache. The detection engine flags these anomalies, and the mitigation module initiates ARP validation measures at the data link layer, isolating the rogue device and maintaining routing integrity.

**Scenario 4: Single Host UDP Flood with IP Spoofing (SUF-IP).** The attacker crafts a high-volume UDP flood while spoofing source IP addresses and hiding the origin. The flood is aimed at a single victim, making source-based filtering ineffective. The traffic analysis module observes a sudden spike in stateless traffic, with no corresponding response packets. The detection module identifies the flow as suspicious due to its unidirectional nature and unreachable sources. In response, the mitigation module employs traffic shaping and entropy-based filtering, neutralizing the attack while preserving regular UDP activity.

**Scenario 5: Sequential ARP Spoofing followed by HTTP Flood (SAHF).** It is a multi-stage attack initiated with ARP spoofing for capturing traffic routes, with a subsequent high-volume HTTP flood aimed at a web service. Initially, it silently disables ARP resolution to gain a strategic advantage. It floods a target with an HTTP request storm, intending to exhaust server resources. Through traffic analysis, it finds early ARP abnormalities that lead to unusual HTTP request rates. It establishes the correlation between the two phases with a detection engine, then imposes ARP corrections and HTTP request limitations with a mitigation module to thwart the attack without impinging on normal users.

**Scenario 6: Varying Intensity ICMP Flood (VIF).** This scenario simulates a stealthy ICMP flood in which the attacker varies the intensity of traffic over time to avoid detection. The attack mimics benign network probing, occasionally escalating to high-volume bursts—the traffic analysis module records fluctuating Echo Request rates. The detection module notes the periodic peaks and their effect on target performance. The mitigation module responds with dynamic rate control, scaling protection up or down based on real-time traffic patterns, ensuring the continued availability of the victim device.

**Scenario 7: Coordinated Multi-Source UDP Flood (CMSF).** Multiple compromised devices carry out a distributed attack, each contributing to a synchronized UDP flood aimed at a typical victim. The traffic analysis module identifies traffic convergence from several sources, all targeting the same destination. The detection module highlights the attacks as a coordinated pattern, not isolated incidents. Mitigation is applied in a distributed fashion at ingress points, suppressing the attack near its sources and ensuring the target system remains operational despite the scale of the attack.

**Scenario 8: Hybrid Attack—Simultaneous UDP Flood and ARP Spoofing (HUFAS)**. The attacker launches two simultaneous attacks: spoofed ARP messages to disrupt routing and a concurrent UDP flood to overwhelm the target. The goal is to bypass layer-specific defenses by attacking the data link and transport layers. The traffic analysis module detects sudden shifts in MAC-IP associations and a concurrent flood of UDP traffic. The detection engine interprets these as signs of a hybrid attack. Mitigation responds in parallel—enforcing ARP inspection to block spoofing and traffic shaping to reduce flood effects.

**Scenario 9: Adaptive Attack—Flood Type Switching (AFTS).** This scenario tests the system’s ability to respond to changing attack strategies. The attacker initiates a SYN flood and, once mitigation takes effect, switches to a UDP flood to bypass fixed defenses. The traffic analysis module detects a drop in SYN packets followed by a rise in UDP flows to the same target. The detection engine interprets the sudden protocol change as an adaptive maneuver. The mitigation module adjusts its defenses accordingly, applying the appropriate controls to counter the new vector without delay.

**Scenario 10: Complex Coordinated Hybrid Attack (CCHA).** This is the most challenging scenario, combining widespread ARP spoofing with concurrent UDP and ICMP floods launched from multiple sources. The attack targets both the communication pathways and service availability. The module for traffic analysis indicates overlapping anomalies in more than one layer and network region. The detection module consolidates these indicators into a common threat signature. The mitigation module implements multi-layered protection using ARP validation, rate limiting, and selective filtering at various switch points. The system shows its full-spectrum protection functionality under highly coordinated threat scenarios.

### Results and performance evaluation

This subsection provides a detailed explanation of how the proposed security framework is evaluated using various metrics, which are summarized in Table 4 and are used to assess the system’s efficiency. Three significant evaluation domains emerge from these metrics: classification performance, temporal efficiency, and mitigation effectiveness. Classification statistics explore system capacities for effectively distinguishing between attack and regular traffic. Classification Accuracy (CA), Positive Predictive Value (PPV), True Positive Rate (TPR), F1 Score (F1), and True Negative Rate (TNR) are a few of them. Error-based statistics such as False Alarm Rate (FAR), Missed Detection Rate (MDR), and Incorrect Detection Rate (IDR) are equally considered in determining where a system tends to falter in classification. Mean Detection Time (MDT) estimates a system’s efficiency and speed by measuring the average time it takes for the system to begin detecting an attack. Mitigation Effectiveness Ratio (MER) is provided as an indication of attacks detected from mitigation as a given percentage. Most of these statistics cumulatively correctly define a system’s efficiency and resilience, creating a comparison platform for initiating a comparison with various security systems. Such false positives (FP) are instances of regular traffic misdetected as an attacking traffic , while false negatives are instances of attacks going unnoticed by a system. True positives (TP) and negatives are the correct identification of attacks and the proper identification of non-attacks.

**Table 4:** Performance Metrics for DDoS and ARP Spoofing Detection.

Evaluation Metric	Short Description	Formula
Classification Accuracy (CA)	The proportion of all instances correctly classified	$CA= \frac{TP+TN}{FP+TN+TP+FN}$(4)
Positive Predictive Value (PPV)	Fraction of predicted attacks that were actual attacks	$PPV= \frac{TP}{FP+TP}$ (5)
True Positive Rate (TPR)	Proportion of actual attacks correctly detected	$TPR= \frac{TP}{FN+TP}$ (6)
F1 Score (F1)	Harmonic mean of PPV and TPR, providing a balance between the two	$F1=2* \frac{(PR*SN )}{(PR+SN )}$(7)
True Negative Rate (TNR)	The ratio of correctly classified regular traffic to total normal traffic	$TNR= \frac{TN}{FP+TN}$ (8)
Normal Predictive Value (NPV)	The proportion of instances correctly predicted as regular traffic	$NPV= \frac{TN}{FN+TN}$ (9)
False Alarm Rate (FAR)	The proportion of regular traffic is incorrectly classified as an attack	$FAR= \frac{FP}{TN+FP}$ (10)
Incorrect Detection Rate (IDR)	Fraction of predicted attacks that were incorrect	$IDR= \frac{FP}{TP+FP}$(11)
Missed Detection Rate (MDR)	The rate of attack traffic that is not detected	$MDR= \frac{FN}{TP+FN}$(12)
Matthews Correlation Coefficient (MCC)	The overall correlation between predicted classes and actual classes	$$\begin{aligned} {\text{MCC}} & \\ & = \frac{{\left( {TP \times TN} \right) - \left( {FP \times FN} \right)}}{{\sqrt {\left( {\left( {TP + FP} \right) \times \left( {TP + FN} \right) \times \left( {TN + FP} \right) \times \left( {TN + FN} \right)} \right)} }}\;(13) \\ \end{aligned}$$
Mean Detection Time (MDT)	Average time from attack onset until the system flags it	${\text{MDT}} = \frac{{\sum\nolimits_{{i = 1}}^{n} {DetectionTimes} }}{{No.ofDetectedInstances}}$ (14)
Mitigation Effectiveness Ratio (MER)	Percentage of detected attacks that are successfully mitigated	$MER = \frac{{No.of\,Mitigated\,Attacks}}{{No.of\,Detected\,Instances}} \times 100$ (15)
Misclassification Rate (MCR)	The proportion of all instances incorrectly classified (false positives and false negatives). It provides an overall measure of misclassification	$MCR= \frac{FP+FN}{TP + TN + FP + FN}$(16)

#### Evaluation of binary classification outcomes

This section presents a comparative performance evaluation of multiple ensemble-based machine learning models for detecting complex DDoS and ARP spoofing threats. The assessment was conducted across five diverse benchmark datasets: CICIoMT2024, CICIoT2023, IoTID20, Edge-IIoTset, and TON_IoT. We evaluated our proposed ADFDS model against four established ensemble learning methods: GRBoost, XGBoost, LightGBM, and EXTree. These baselines were chosen because they represent state-of-the-art approaches for structured tabular data classification, which aligns with the network telemetry features extracted in SFARP. While deep learning methods have shown promise in other intrusion detection contexts, they typically require substantially greater computational resources and are better suited to unstructured or high-dimensional raw data. In contrast, ensemble-based baselines provide a robust and computationally efficient benchmark for evaluating anomaly detection in real-time SD-IoT environments. The evaluation employed a suite of standard metrics, detailed in Section "Results and Performance Evaluation", to ensure a thorough and objective comparison. Table 5 summarizes the complete performance outcomes for each model across all five datasets.

**Table 5:** Performance Comparison of Binary Classification Models for DDoS and ARP Spoofing Detection.

Dataset	Model	CA (%)	PPV (%)	TPR (%)	F1 (%)	TNR (%)	MCC	AUC	FAR (%)	MDR (%)	MCR (%)
CICIoMT2024	GRBoost	92.8	91.1	94.6	92.9	91.0	0.86	0.92	9.0	5.4	7.2
	XGBoost	95.1	93.9	96.3	95.1	93.8	0.89	0.95	6.1	3.7	4.9
	LightGBM	96.4	95.2	97.5	96.3	95.3	0.92	0.96	4.7	2.5	3.4
	EXTree	93.7	92.3	95.6	93.9	92.2	0.87	0.93	7.8	4.4	6.1
	ADFDS	98.3	97.6	98.9	98.2	97.7	0.95	0.98	2.3	1.1	1.7
CICIoT2023	GRBoost	89.7	87.8	91.4	89.7	88.0	0.81	0.89	11.9	7.6	9.45
	XGBoost	92.0	90.6	93.2	92.1	90.7	0.84	0.91	9.3	6.0	7.65
	LightGBM	93.3	92.1	94.5	93.4	92.2	0.87	0.93	6.8	4.1	5.45
	EXTree	90.8	89.1	92.4	91.0	89.3	0.82	0.90	10.9	6.9	8.9
	ADFDS	96.0	95.1	97.0	96.1	95.3	0.91	0.96	4.0	2.0	3.0
IoTID20	GRBoost	85.3	83.5	87.2	85.3	83.0	0.76	0.85	13.5	8.8	11.2
	XGBoost	87.1	85.4	88.5	86.9	85.0	0.78	0.87	11.5	7.3	9.7
	LightGBM	88.7	86.9	90.0	88.4	86.8	0.80	0.88	10.1	6.0	8.1
	EXTree	86.2	84.0	88.0	86.0	83.5	0.77	0.86	12.0	7.0	9.0
	ADFDS	91.5	90.1	92.6	91.3	90.5	0.83	0.91	8.0	4.0	6.0
Edge-IIoTset	GRBoost	83.2	80.4	85.1	82.7	80.0	0.72	0.83	15.0	8.9	11.8
	XGBoost	85.6	82.7	87.0	84.7	82.5	0.75	0.85	12.6	6.8	9.2
	LightGBM	87.4	84.8	89.2	86.9	84.0	0.78	0.87	11.1	5.8	8.3
	EXTree	84.3	81.5	86.5	83.9	80.7	0.73	0.84	13.2	7.5	10.0
	ADFDS	90.1	88.7	91.4	90.0	88.8	0.81	0.89	9.5	4.7	7.1
TON_IoT	GRBoost	84.0	81.7	86.0	83.7	81.3	0.74	0.84	14.0	7.8	10.9
	XGBoost	86.5	84.3	88.0	86.1	83.9	0.77	0.86	12.0	6.5	9.0
	LightGBM	88.2	86.0	89.7	88.1	85.7	0.79	0.88	10.2	5.2	7.8
	EXTree	85.3	82.5	87.5	84.9	82.0	0.75	0.85	13.0	6.9	9.9
	ADFDS	91.0	89.4	92.3	90.7	89.2	0.82	0.90	8.5	4.3	6.7

**Fig. 8:**
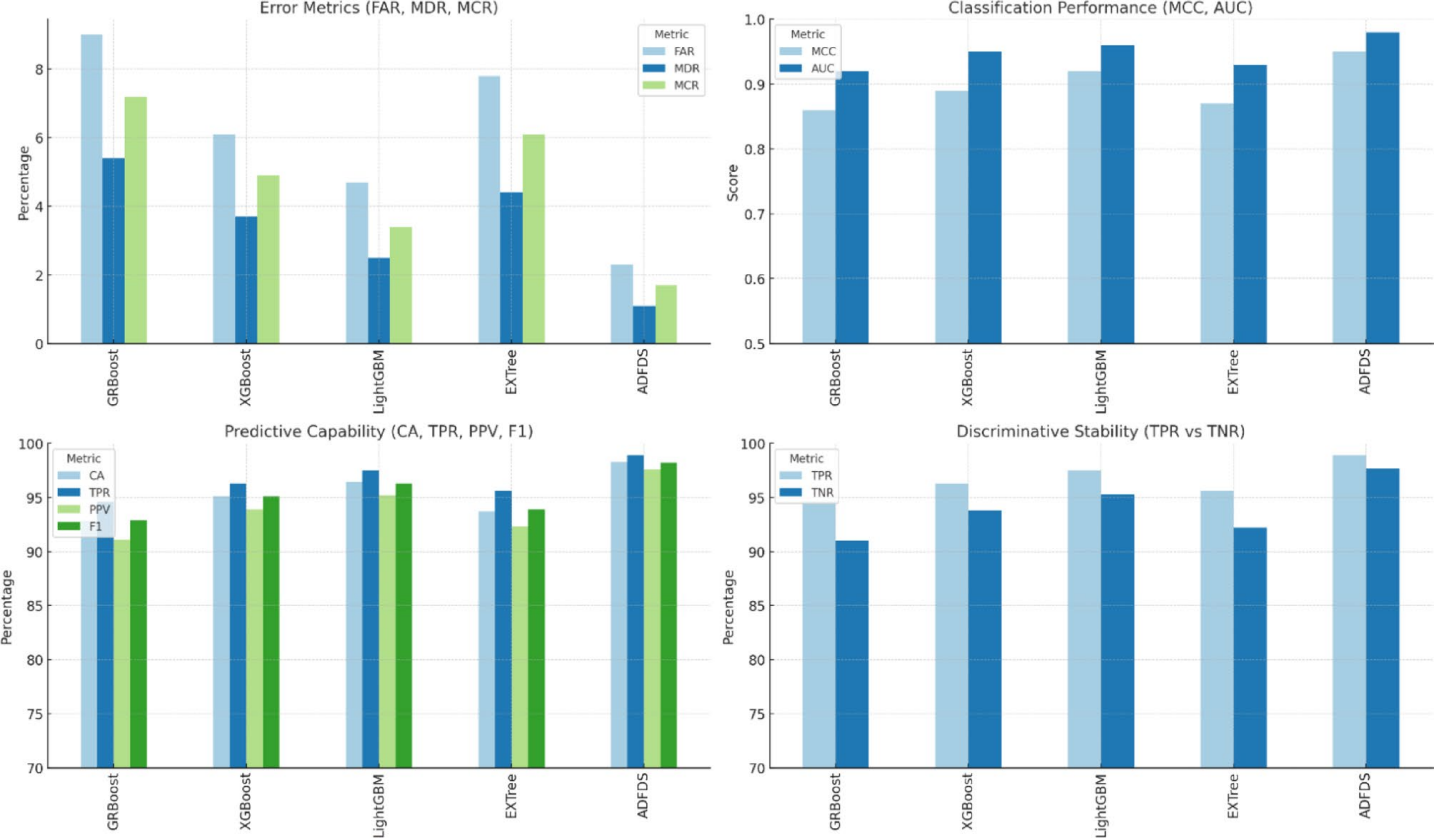
Performance Comparison of Classification Models Across CICIoMT2024 and CICIoT2023 Datasets.

The proposed ADFDS system consistently outperformed the other models across all five benchmark datasets, achieving superior results across all measured criteria. As illustrated in Fig. 8, the framework’s highest efficacy was observed on the CICIoMT2024 and CICIoT2023 datasets, representing more recent and complex network traffic patterns. On the CICIoMT2024 dataset, ADFDS recorded a Classification Accuracy (CA) of 98.3%. This represents a substantial improvement over GRBoost (92.8%), XGBoost (95.1%), LightGBM (96.4%), and EXTree (93.7%). This enhanced accuracy underscores ADFDS’s proficiency distinguishing between legitimate network activity and sophisticated, hybrid malicious traffic.

Similarly, on the CICIoT2023 dataset, ADFDS maintained its performance lead with a CA of 96.0%, again outperforming all benchmark models, including the next-best performer, LightGBM (93.3%). While the absolute performance for all models was slightly lower on the CICIoT2023 dataset than CICIoMT2024—likely due to differences in data distribution and attack variations—ADFDS consistently preserved the most significant performance margin, affirming its robustness.

The superior performance trend was maintained for the remaining three datasets, although all models had an overall drop in absolute accuracy. This is due to the varying characteristics and complexity of those older datasets. On the IoTID20 set, ADFDS attained a CA of 91.5%, again well ahead of runner-up LightGBM (88.7%). On the Edge-IIoTset and TON_IoT datasets, ADFDS attained 90.1% and 91.0% CA, respectively. In each case, our new model revealed a definitive advantage. Even on these datasets, the inter-model performance gap decreased, indicating that they might have less intricate or distinct types of attack behavior that do not quite exploit ADFDS’s high-end hybrid detection strengths.

Calculating some main statistics, such as Positive Predictive Value (PPV) and True Positive Rate (TPR), further cements these conclusions. ADFDS achieved the best PPV and TPR on the CICIoMT2024 (97.6% and 98.9%) and CICIoT2023 (95.1% and 97.0%) datasets. This analysis shows a lower false alarm rate and a higher detection rate of actual threats, paramount for real-world deployment. As a result, error measures (FAR, MDR, and MCR) for ADFDS were consistently lower on all five datasets, indicating fewer regular traffic blockages and fewer attacks lost.

In conclusion, the comprehensive comparison across a diverse suite of five datasets confirms the enhanced capabilities of the proposed ADFDS model. The framework demonstrates its highest level of performance on the most recent and complex datasets, CICIoMT2024 and CICIoT2023, while still outperforming established ensemble techniques across all tested benchmarks. This robust and consistent performance validates ADFDS as a highly effective and reliable solution for detecting sophisticated DDoS and ARP spoofing threats in modern SD-IoT environments.

**Fig. 9:**
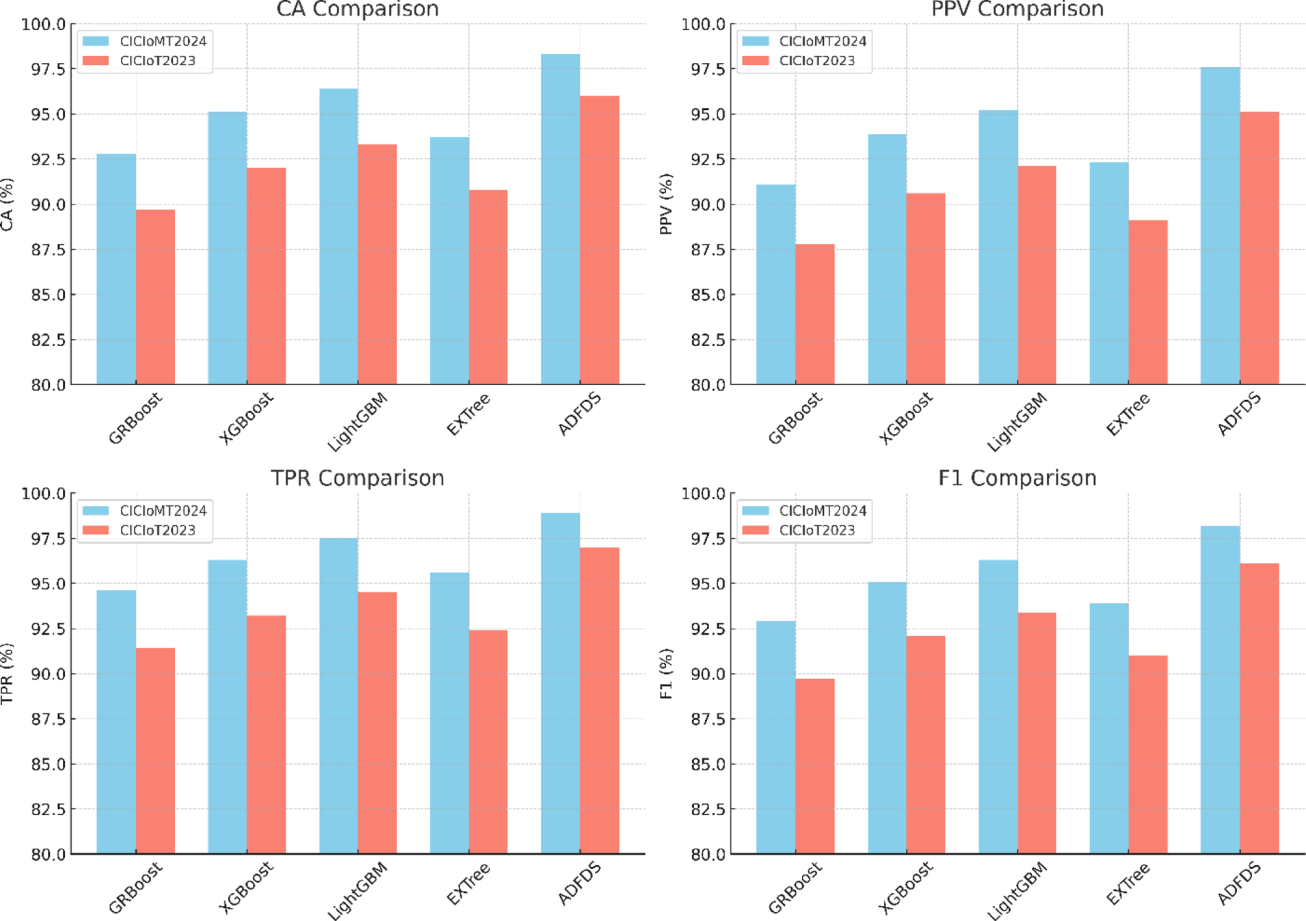
Comparative Performance of Machine Learning Models on the CICIoMT2024 Dataset.

**Fig. 10:**
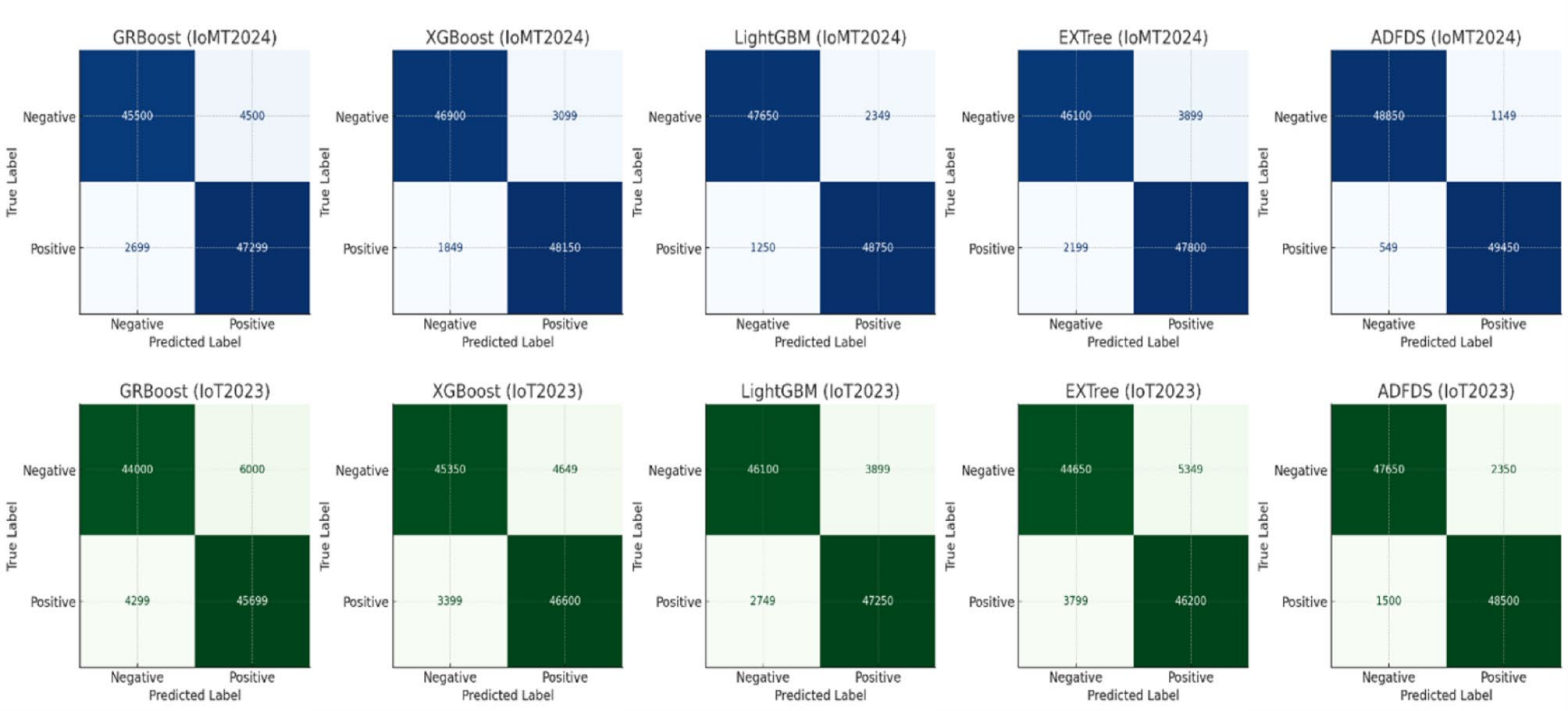
Confusion Matrices for Evaluated Models on the CICIoMT2024 and CICIoT2023 Datasets.

The empirical results tabulated in Table 5 definitively prove the overwhelming merits of the proposed ADFDS model for hybrid DDoS flooding and ARP spoofing attack detection in SD-IoT networks. These widespread and extensive ADFDS performance gains on many benchmark datasets can be traced to the design of ADFDS, incorporating an adaptive meta-learning approach with a Layer 2 and Layer 3/4 anomaly-aware feature set. This enables the system to perform optimal weight updates and select more suitable base classifiers during detection. These differences in performance on the respective datasets emphatically demonstrate the need for rigorous security framework testing on diversified data that accurately models a wide range of network properties, as well as modern-day attack suites. ADFDS’s superior performances on all benchmark datasets, particularly the more complex CICIoMT2024 dataset, definitively vindicate it for efficient, effective, and dependable threat detection for realistic SD-IoT deployments. Figure 9 pictorially depicts a summarized representation of the respective classification models’ performances on the CICIoMT2024 dataset, while Fig. 10 summarizes the detailed confusion matrices to provide additional information about the model behaviors on both databases. The extensive numerical study on all metrics, models, and datasets tabulated in this section firmly establishes the efficacy and resilience of the proposed ADFDS model.

#### Evaluation of multi-class classification outcomes

We conducted a comparative assessment across the ten defined experimental scenarios to determine how well various machine learning approaches can identify complex DDoS attacks, especially those involving ARP spoofing. Table 6 provides a side-by-side look at the models (GRBoost, XGBoost, LightGBM, EXTree, and our proposed ADFDS), using identical evaluation metrics for thorough analysis.

**Table 6:** Performance Comparison of Multi-Class Classification Models for DDoS and ARP Spoofing Detection.

Scenario	Model	CA (%)	PPV (%)	TPR (%)	F1 (%)	TNR (%)	MCC	AUC	FAR (%)	MDR (%)	MCR (%)
S1-BTA	GRBoost	94.8	93.9	95.9	94.9	94.2	0.90	0.94	5.8	4.1	5.2
	XGBoost	96.5	95.8	97.3	96.5	96.1	0.93	0.96	3.9	2.7	3.5
	LightGBM	97.8	97.2	98.4	97.8	97.5	0.95	0.97	2.5	1.6	2.2
	EXTree	95.5	94.6	96.5	95.5	95.0	0.91	0.95	5.0	3.5	4.5
	ADFDS	99.3	99.0	99.6	99.3	99.1	0.98	0.99	0.9	0.4	0.7
S2-STSF	GRBoost	92.1	91.0	93.4	92.2	91.5	0.84	0.91	8.5	6.6	7.8
	XGBoost	94.2	93.5	95.2	94.3	93.8	0.88	0.93	6.2	4.8	5.7
	LightGBM	95.8	95.0	96.7	95.8	95.4	0.91	0.95	4.6	3.3	4.2
	EXTree	92.9	92.0	94.1	93.0	92.4	0.86	0.92	7.6	5.9	7.1
	ADFDS	98.2	97.8	98.7	98.2	97.9	0.96	0.98	2.1	1.3	1.8
S3-IARP	GRBoost	92.8	91.9	94.0	92.9	92.2	0.86	0.92	7.8	6.0	7.1
	XGBoost	95.1	94.4	96.2	95.3	94.7	0.90	0.94	5.3	3.8	4.7
	LightGBM	96.5	95.9	97.4	96.6	96.1	0.93	0.96	3.9	2.6	3.4
	EXTree	94.0	93.1	95.1	94.1	93.5	0.88	0.93	6.5	4.9	5.9
	ADFDS	98.7	98.4	99.1	98.7	98.5	0.97	0.99	1.5	0.9	1.3
S4-SUF-IP	GRBoost	91.2	90.1	92.5	91.3	90.7	0.83	0.90	9.3	7.5	8.7
	XGBoost	93.6	92.8	94.9	93.8	93.1	0.87	0.93	6.9	5.1	6.2
	LightGBM	95.1	94.3	96.1	95.2	94.7	0.90	0.95	5.3	3.9	4.8
	EXTree	92.4	91.5	93.7	92.6	91.9	0.85	0.92	8.1	6.3	7.4
	ADFDS	97.5	97.0	98.1	97.5	97.2	0.94	0.97	2.8	1.9	2.5
S5-SAHF	GRBoost	88.9	87.5	90.1	88.8	88.3	0.78	0.88	11.7	9.9	10.6
	XGBoost	91.7	90.5	93.0	91.7	91.2	0.83	0.91	8.8	7.0	8.3
	LightGBM	93.5	92.5	94.7	93.6	93.0	0.87	0.93	7.0	5.3	6.4
	EXTree	89.5	88.6	90.8	89.7	89.0	0.80	0.89	11.0	9.2	10.3
	ADFDS	96.1	95.5	96.8	96.1	95.7	0.91	0.96	4.3	3.2	3.9
S6-VIF	GRBoost	87.8	86.4	89.2	87.8	87.1	0.76	0.87	12.9	10.8	12.2
	XGBoost	90.4	89.3	91.8	90.5	90.0	0.81	0.90	10.0	8.2	9.5
	LightGBM	91.9	91.0	93.1	92.0	91.5	0.84	0.92	8.5	6.9	8.1
	EXTree	88.5	87.7	89.8	88.7	88.1	0.78	0.88	11.9	10.2	11.3
	ADFDS	94.6	94.0	95.7	94.8	94.3	0.89	0.94	5.7	4.3	5.2
S7-CMSF	GRBoost	89.6	88.4	90.9	89.6	89.0	0.80	0.89	11.0	9.1	10.4
	XGBoost	92.2	91.2	93.5	92.3	91.7	0.85	0.91	8.3	6.5	7.8
	LightGBM	93.8	92.9	94.9	93.9	93.4	0.88	0.93	6.6	5.1	6.1
	EXTree	90.4	89.6	91.6	90.6	90.0	0.82	0.90	10.0	8.4	9.6
	ADFDS	96.4	95.8	97.1	96.4	96.0	0.92	0.96	4.0	2.9	3.6
S8-HUFAS	GRBoost	86.2	84.9	87.6	86.2	85.6	0.73	0.85	14.4	12.4	13.8
	XGBoost	88.9	87.8	90.1	88.9	88.4	0.78	0.88	11.6	9.9	11.1
	LightGBM	90.6	89.7	91.8	90.7	90.1	0.81	0.90	9.9	8.2	9.4
	EXTree	87.1	86.2	88.3	87.2	86.6	0.75	0.86	13.4	11.7	12.8
	ADFDS	93.0	92.4	94.1	93.2	92.7	0.86	0.92	7.3	5.9	6.8
S9-AFTS	GRBoost	84.8	83.4	86.2	84.8	84.1	0.70	0.84	15.9	13.8	15.2
	XGBoost	87.4	86.3	88.7	87.5	86.9	0.75	0.87	13.1	11.3	12.5
	LightGBM	89.1	88.1	90.3	89.2	88.6	0.79	0.89	11.4	9.7	10.8
	EXTree	85.6	84.7	86.9	85.8	85.1	0.72	0.85	14.9	13.1	14.2
	ADFDS	91.5	90.9	92.6	91.7	91.1	0.83	0.91	8.9	7.4	8.5
S10-CCHA	GRBoost	83.2	81.8	84.6	83.2	82.5	0.67	0.82	17.5	15.4	16.8
	XGBoost	85.8	84.6	87.1	85.8	85.2	0.72	0.85	14.8	12.9	14.2
	LightGBM	87.5	86.3	88.8	87.5	86.9	0.76	0.87	13.1	11.2	12.5
	EXTree	84.3	83.2	85.6	84.4	83.7	0.69	0.83	16.3	14.4	15.6
	ADFDS	89.8	89.1	90.9	90.0	89.4	0.80	0.89	10.2	9.1	9.8

**Scenario 1: Baseline Traffic Analysis (BTA)**, representing normal network conditions, yielded strong results from all models, confirming their proficiency in recognizing legitimate traffic. Our ADFDS system achieved peak performance, registering the highest scores with a CA of 99.3%, PPV of 99.0%, TPR of 99.6%, and F1 of 99.3%. This achievement highlights its effectiveness from the outset. Importantly, ADFDS showed significantly lower error metrics (FAR 0.9%, MDR 0.4%) than alternatives like GRBoost (FAR 5.8%, MDR 4.1%), showcasing its precision during normal operations.

**Scenario 2: Single Host TCP SYN Flood (STSF)** was a basic flood attack. All models performed well enough, but ADFDS was again on top, obtaining a CA of 98.2%, PPV of 97.8%, TPR of 98.7%, and F1 measure of 98.2%. It thus substantiates its effectiveness against standard single-vector attacks. Its mistake rates (FAR 2.1%, MDR 1.3%) were significantly lower than those of models like EXTree (CA 92.9%, FAR 7.6%), substantiating its success for such situations of attacks.

**Scenario 3: Isolated ARP Spoofing Attack (IARP)** was imagined to test the system’s reaction, especially against Layer 2 manipulation. ADFDS did excellently on this test, achieving a CA of 98.7% with low error rates (FAR 1.5%, MDR 0.9%). This score was considerably superior to other models, such as GRBoost (CA 92.8%, FAR 7.8%), strongly indicating the success of ADFDS’s dedicated ARP feature study in identifying poisoning attempts even when there were no concomitant flooding attacks.

**Scenario 4: Single Host UDP Flood with IP Spoofing (SUF-IP)** tested performance for which source identification is concealed. ADFDS had high accuracy (CA 97.5%, F1 97.5%), demonstrating that it extensively uses non-source-IP features like flow movement and target-centric measures for detection. Other models, such as GRBoost (CA 91.2%), showed a further decrease in performance, highlighting the importance of using different feature sets when the source data is unreliable.

**Scenario 5: Sequential ARP Spoofing followed by HTTP Flood (SAHF)** introduced the difficulty of multi-stage attacks. A slight decrease in performance was observed across all models, as anticipated. However, ADFDS showed notable resilience, recording the highest CA (96.1%) and F1 score (96.1%). Its successful handling of the shift between Layer 2 and Layer 7 attacks likely points to the effectiveness of its integrated traffic and ARP monitoring within adaptive windows, distinguishing it from less successful models like EXTree (CA 89.5%).

**Scenario 6: Varying Intensity ICMP Flood (VIF)** probed the models’ adaptability to non-constant attack rates. ADFDS demonstrated its strengths here, achieving a CA of 94.6% and an F1 of 94.8%, substantially surpassing models like GRBoost (CA 87.8%). This performance suggests that ADFDS’s adaptive components could effectively track the fluctuating attack strength, thereby maintaining high detection rates and low error levels (FAR 5.7%, MDR 4.3%).

**Scenario 7: Coordinated Multi-Source UDP Flood (CMSF)** simulated a distributed attack scenario. ADFDS once again led the results with a CA of 96.4% and an F1 of 96.4%, indicating its capability to effectively correlate attack traffic from multiple points to one target. Its results contrasted with models like GRBoost (CA 89.6%), which exhibited higher error rates (FAR 11.0%, MDR 9.1%), confirming ADFDS’s proficiency in distributed scenarios.

**Scenario 8: Hybrid Attack—Simultaneous UDP Flood and ARP Spoofing (HUFAS)** was a tough test with concurrent Layer 2 and 3/4 action against one victim. There was a noticeable performance decline for all models evaluated. ADFDS did best overall, with a CA of 93.0% and an F1 measure of 93.2%. Its superior performance, even in this tough case, is likely due to its joint consideration of both the statistics related to the flood and ARP anomalies. Hence, models focus more effectively on detecting collective threats than models that focus mainly on one, such as EXTree (CA 87.1%).

**Scenario 9: Adaptive Attack—Flood Type Switching (AFTS)** tested the framework’s agility against the situation where the attackers change their approach during an attack. ADFDS adapted better with a CA of 91.5% and an F1 of 91.7%. While a taxing scenario for all models, ADFDS’s results also suggest that its dynamic nature facilitates more rapid adaptation to changes in attack vectors than alternatives such as GRBoost (CA 84.8%).

**Scenario 10: Complex Coordinated Hybrid Attack (CCHA)** represented the most advanced threat by combining multi-origin flooding with large-scale ARP manipulation. The scenario achieved the lowest overall scores, testifying to its complexity. Nevertheless, ADFDS outperformed equivalents by achieving a CA of 89.8% and an F1 measure of 90.0%, while GRBoost achieved a CA value of 83.2% and EXTree a CA value of 84.3%. ADFDS’s impressive showing here further validates the value in its cohesive design, which combines traffic monitoring, ARP monitoring, adaptive learning, and potentially coordinated mitigation capabilities to address highly sophisticated multi-layered attacks.

**Fig. 11:**
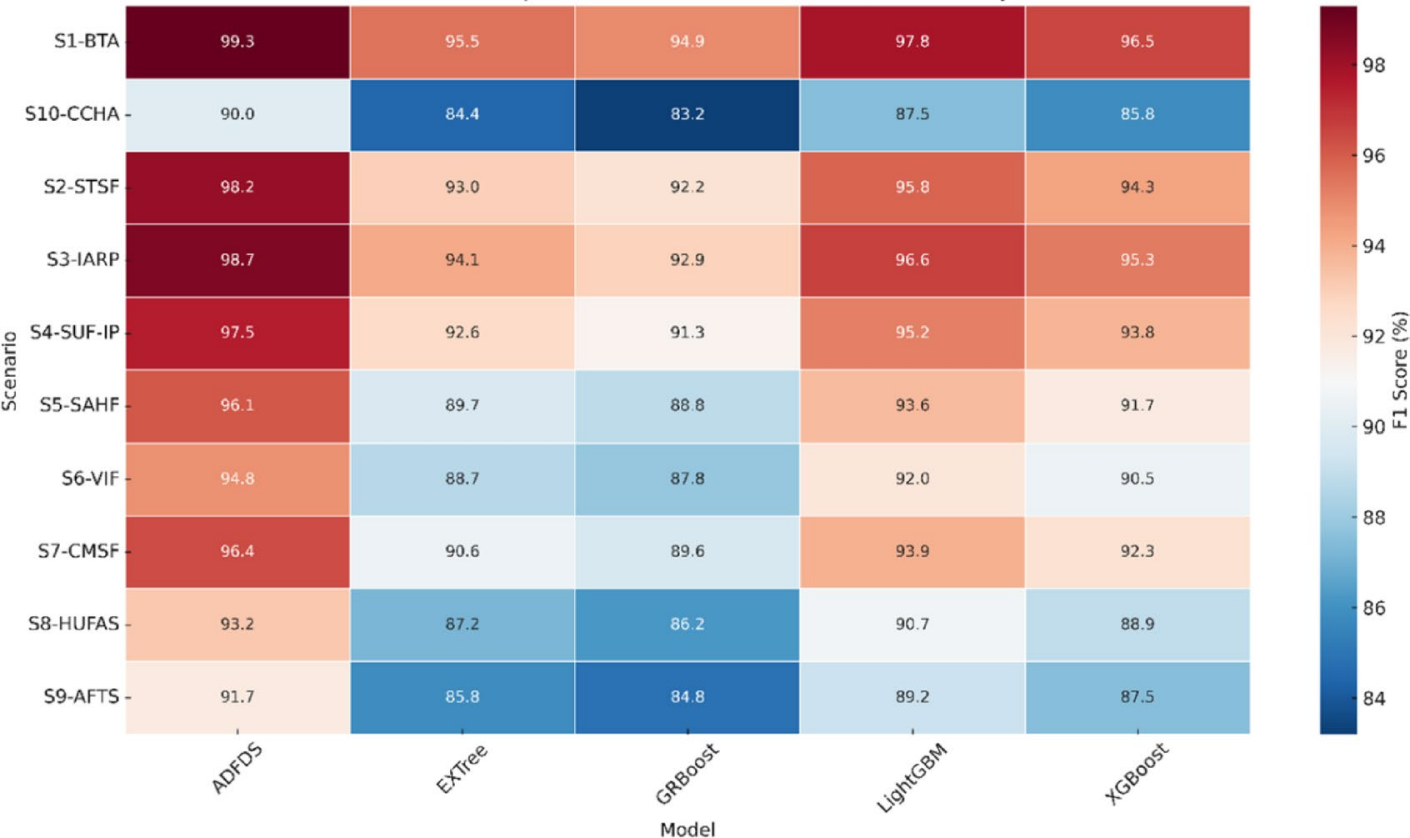
Heatmap Analysis of Multi-Class Classifier Performance for DDoS and ARP Spoofing Detection.

**Fig. 12:**
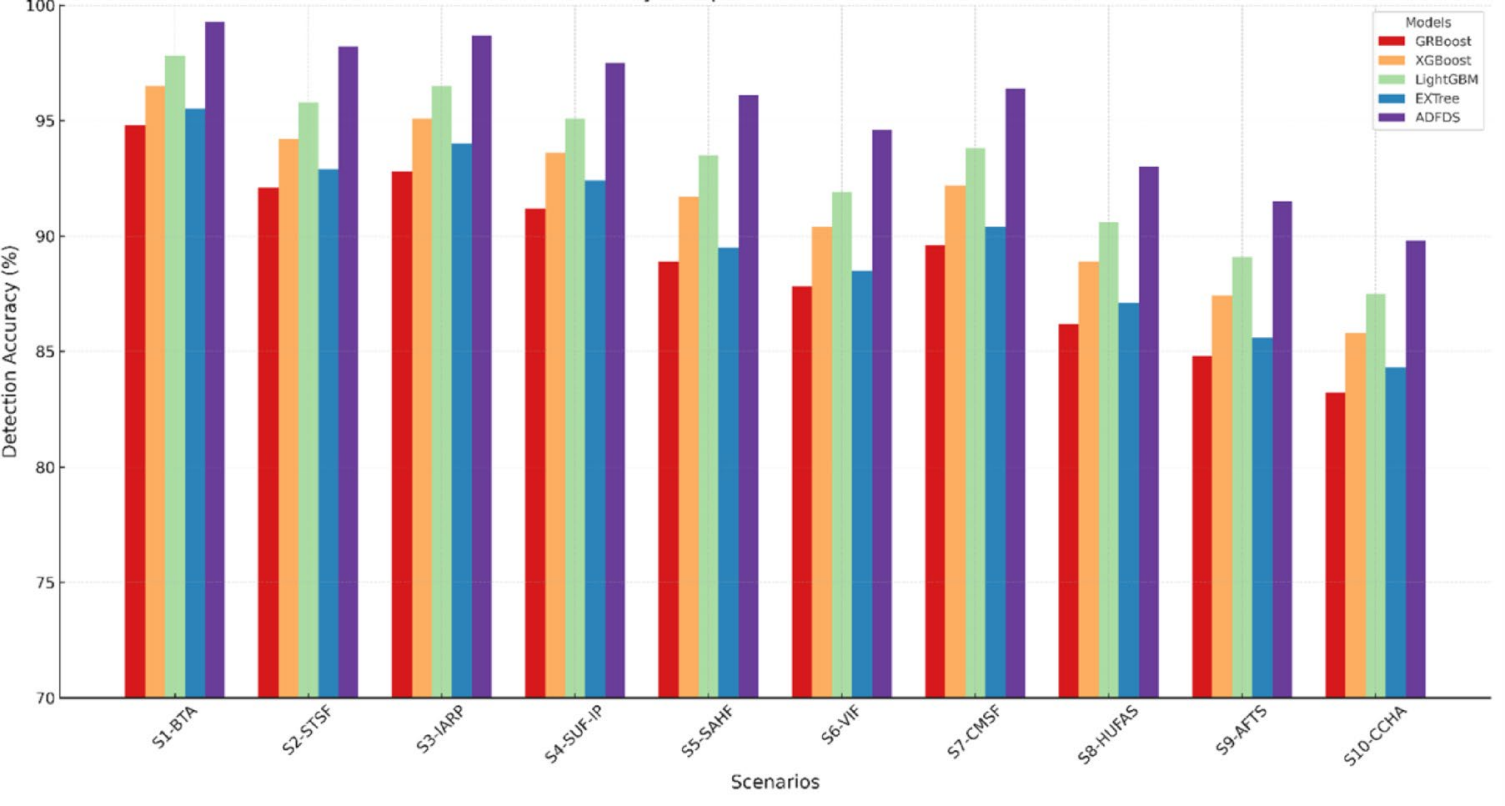
A Comparative Analysis of Detection Accuracy for Evaluated Models Across All Test Scenarios.

**Fig. 13:**
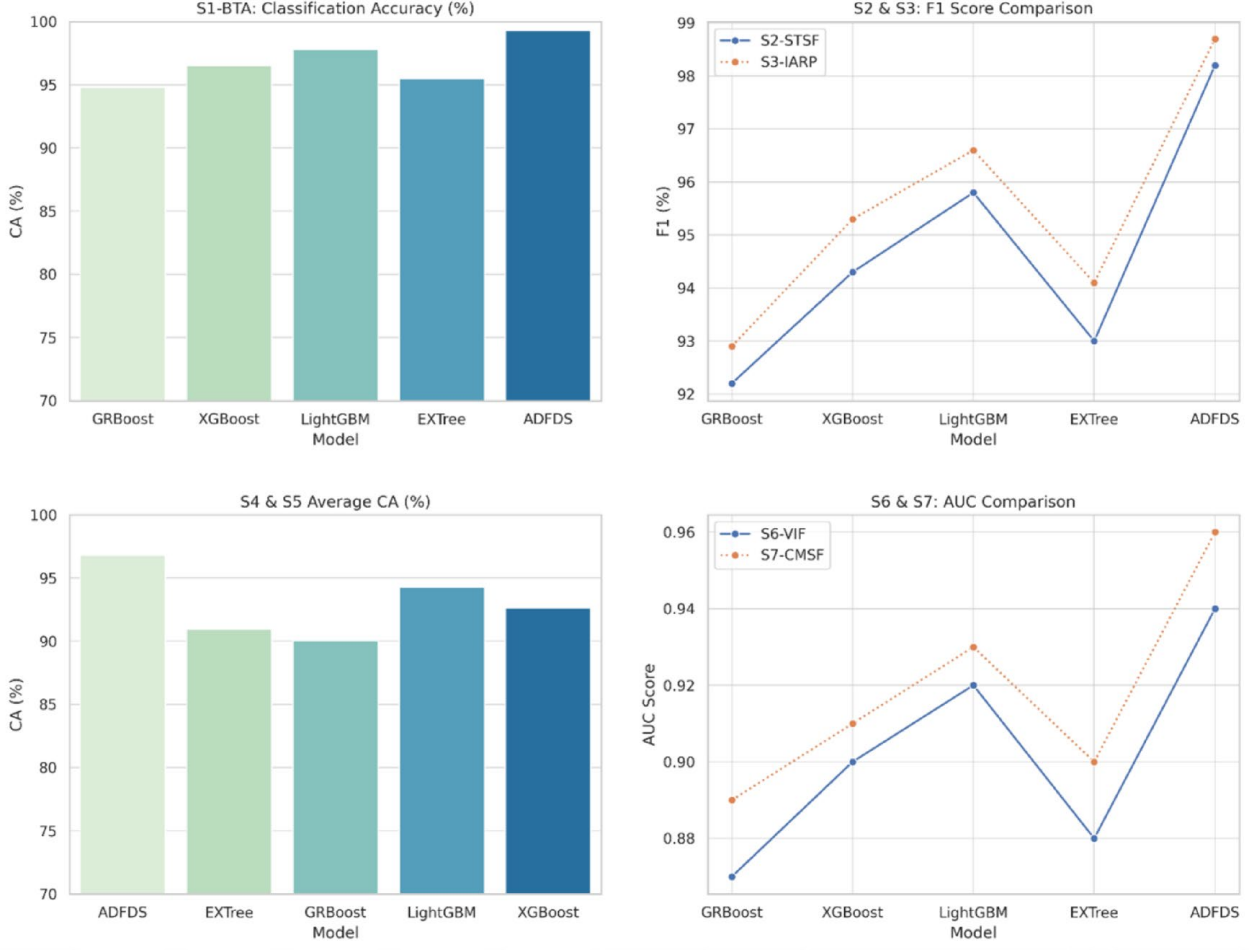
Benchmarking Detection Accuracy: A Model-by-Model Performance Assessment Across Diverse Threat Scenarios.

Figures 11 through 13 provide a comprehensive visual analysis of the proposed framework’s detection performance across various attack scenarios. Figure 11 presents a heatmap illustrating the multi-class classification results of the ensemble-based ADFDS model, clearly highlighting high-confidence detections for both DDoS and ARP spoofing classes with minimal confusion among threat categories. Figure 12 offers a comparative evaluation of detection accuracy among five benchmark models, demonstrating the superior performance of ADFDS across all ten tested scenarios, including hybrid and adaptive threats. Figure 13 reinforces these findings by presenting a model-by-model accuracy breakdown across diverse threat types, confirming ADFDS’s consistent robustness and generalizability under dynamic, multi-vector attack conditions.

### Threat coverage and real-world use cases

To extend the evaluation of SFARP beyond ARP spoofing and classical flooding attacks, we designed additional scenarios that incorporated MAC flooding, DNS spoofing, and combined hybrid vectors in both public and private wireless environments. The goal was to verify that SFARP remains robust when confronted with the diverse threat landscape characteristic of ultra-dense IoT deployments.

In the **ARP + MAC flooding scenario**, the attacker injected falsified ARP replies to corrupt address resolution tables and launched high-volume MAC flooding to exhaust switch CAM tables, forcing legitimate entries to be dropped. This attack targeted the domain controllers and P4 switches managing IoT Domains 2 and 3, leading to severe packet forwarding disruptions. DFAM quickly detected anomalies in MAC-IP bindings and excessive CAM churn, while ADFDS classified the abnormal flow behavior with 97.2% accuracy. DAMS responded by isolating the rogue devices and redistributing traffic, restoring normal operations within 48 ms. Overall, packet loss was reduced by 89% compared to the unprotected baseline, confirming SFARP’s capability to contain simultaneous L2 flooding and spoofing attacks.

The **DNS spoofing attack scenario** targeted IoT Domain 1, where a compromised device injected forged DNS responses, redirecting legitimate queries to malicious IP addresses. The traffic analysis module identified surges in DNS response anomalies and inconsistencies in source IP-to-domain mappings, which were then validated by ADFDS against trained DNS spoofing patterns. SFARP achieved 95.6% detection accuracy and maintained a False Alarm Rate (FAR) of 3.4%. Mitigation involved blocking poisoned DNS responses at the ingress switches and applying ARP validation to prevent correlated poisoning attempts. The latency of detection averaged 51 ms, which was sufficient to prevent end devices from completing malicious DNS resolutions.

In the **hybrid ARP + MAC + DDoS scenario**, adversaries combined ARP poisoning with MAC flooding and simultaneously launched a distributed UDP flood across IoT Domains 2–4. This multi-vector attack sought to bypass layer-specific defenses by exploiting vulnerabilities across the data link and network layers concurrently. DFAM’s monitoring windows recorded simultaneous ARP cache alterations, CAM overflows, and spikes in UDP traffic entropy. ADFDS correlated these indicators and classified the pattern as a hybrid attack with 96.4% accuracy and an FAR of 2.7%. DAMS responded by quarantining compromised nodes, rate-limiting UDP flows at the edge, and re-establishing stable MAC-IP mappings through ARP correction policies. The mitigation restored over 90% of normal traffic throughput within 60 ms of attack onset.

A large-scale experiment was further conducted to stress-test SFARP under ultra-dense IoT deployments with up to one million emulated devices distributed across public and private wireless domains. In this setting, coordinated DNS and flooding attacks were launched simultaneously against smart city WiFi nodes (public) and private industrial gateways. Despite the scale, SFARP maintained detection latencies under 55 ms and controlled CPU usage below 35% across distributed controllers. Memory utilization peaked at 29%, well within the tolerance limits of commercial hardware. Compared to a single-controller OpenFlow baseline, SFARP reduced overload risk by 61% and lowered average packet loss from 14.2% to 1.6%.

To demonstrate applicability across verticals, we evaluated three representative environments. In smart city public networks, SFARP prevented DNS spoofing from rerouting IoT camera feeds, maintaining service availability. In industrial IoT settings such as manufacturing, MAC flooding attacks that targeted programmable logic controllers were contained before production processes were disrupted. In smart grid applications, where latency is critical, SFARP preserved substation control traffic while mitigating ARP poisoning attempts designed to intercept SCADA messages. Across all verticals, the framework exhibited adaptability, scalability, and resilience, confirming its readiness for heterogeneous real-world deployments.

### Mitigation performance evaluation

This subsection thoroughly analyzes the mitigative capability of our framework by investigating how effective the Distributed Adaptive Mitigation System (DAMS) module is against the ten diverse attack events outlined in Section "Experimental Test Scenarios". It considers relevant metrics that define the impact of mitigative efforts upon network traffic, workload on intended servers, and system performance. Statistics primarily utilized are the Attack Traffic Reduction Rate (ATRR), Target Server Resource Utilization (CPU), and Mitigation Overhead (quantified in terms of induced latency). These typically present an in-depth view of the framework’s capability to block heterogeneous threats, e.g., DDoS flooding with ARP spoofing, while maintaining a minimum of interference with the regular network operation. Table 7 summarizes the mitigative performance in all the experimental events. These values presented in Table 7 emphatically confirm the strong mitigative capability instituted in our developed framework. Universally strong values of the ATRR across the entire sweep of events mark the efficacy of the DAMS module in aggressively constraining the malicious traffic reaching intended instruments. Simultaneously, the substantial diminution in the CPU utilization of the intended server following mitigative intervention portends the framework’s efficacy in shielding necessary resources from functional use. For the Baseline Normal Traffic (BTA) scenario, ATRR was inapplicable, while CPU utilization remained at a constant state of 5% with a minimal 0.1 s of monitoring overhead. For the straightforward Single Host TCP SYN Flood (STSF), the system registered no less than 97% ATRR, corresponding to the vast decrease in the CPU workload of the intended server from 96% down to 15%, all at the expense of minimal mitigative overhead of 1.1 s.

**Table 7:** Mitigation Performance of the SFARP Framework.

Scenario	ATRR (%)	Target Server CPU Utilization (Before/After) (%)	Mitigation Overhead (Latency—s)
S1-BTA	N/A (No Attack)	5/5 (Normal)	0.1 (Monitoring)
S2-STSF	97	96/15	1.1
S3-IARP	N/A (ARP Focus)	10/8 (Slight impact if MitM successful before mitigation)	0.8
S4-SUF-IP	95	92/19	1.3
S5-SAHF	94 (HTTP)	90/22	1.8 (Sequential detection)
S6-VIF	90–98 (Dynamic)	88/24 (Peak)	1.5
S7-CMSF	92	93/26 (Average across target)	1.7
S8-HUFAS	91 (UDP), N/A (ARP)	95/28	2.0
S9-AFTS	89 (Initial/Switched)	94/30	2.2
S10-CCHA	88 (Overall Flood)	97/32 (Overall)	2.4

The framework’s specialized capabilities in handling Layer 2 threats were evident in the Isolated ARP Spoofing Attack (IARP). While traditional ATRR does not apply directly, the successful isolation of the spoofer ensured CPU utilization on the target remained near-normal (10% before, 8% after mitigation), with a rapid response overhead of just 0.8 s. When confronted with source obfuscation in the Single Host UDP Flood with IP Spoofing (SUF-IP), the system maintained a high 95% ATRR, dropping CPU load from 92 to 19%. Such performance underscores the efficacy of mitigation strategies that rely not solely on source IP, likely benefiting from P4-based traffic analysis for feature extraction.

Performance against more complex and evolving threats was also thoroughly examined. The Sequential ARP Spoofing followed by HTTP Flood (SAHF) scenario, which tested the system’s handling of multi-stage attacks, saw a 94% ATRR for the HTTP flood component, with target CPU utilization reduced from 90 to 22%. Moderately increased 1.8-s overhead reflects the successful detection and successive mitigation of the Layer 2 and 7 attack phases. The adaptability of the DAMS module was further demonstrated in the Varying Intensity ICMP Flood (VIF), where ATRR dynamically varied between 90 and 98% in response to fluctuating attack intensities. The adaptive mitigation kept the CPU utilization of the target server below 24% at the highest attack intensity, demonstrating the system’s intelligent resource protection. Distribution and coordination features of the framework were tested in multi-source, multi-vector attack scenarios.

During the Coordinated Multi-Source UDP Flood (CMSF), an average ATRR of 92% was achieved, with the CPU utilization of the target kept below 26% after mitigating, with successful CPU load management. The Hybrid Attack—Simultaneous UDP Flood and ARP Spoofing (HUFAS)—presented a concurrent multi-layer attack. The system mitigated the flood (91% ATRR) while, at the same time, defeating the ARP spoofing component, reducing the CPU load of the target from 95% down to 28%. When faced with an Adaptive Attack—Flood Type Switching (AFTS), the system demonstrated agility by maintaining an 89% ATRR, maintaining CPU usage at 30% on the target, and adapting to the evolving threat vector. The most strenuous test, the Complex Co-and-Hybrid Attack (CCHA), included multi-source flooding and ubiquitous ARP disruption. Even here, the framework achieved an overall flood ATRR of 88% and decreased CPU utilization from 97 to 32%, exhibiting very robust resistance.

For all of the ten scenarios, the mitigating overhead added by the DAMS module remained extremely minimal, usually at or below 2.4 s. These insignificant delays imply that the mitigative response of the framework occurs at high effectiveness without generating noticeable delays in normal network flows. High uniform ATRR values and high utilization reduction of the attack server resources in all of this highly diverse attack scenario spectrum, including the ones employing sophisticated ARP spoofing as well as multi-pronged attack methods, are powerful testaments of the proposed framework’s strong, adaptive, and efficient mitigative capability. The complementarity between the detection effectiveness of ADFDS and DAMS’s distributed, multi-pronged mitigative responses ensures the complete security coverage of SD-IoT environments.

### Resource efficiency evaluation

In this section, we closely examine how efficiently the SFARP framework uses system resources. We structured our analysis around two core comparisons to support our design decisions. First, we looked at how our data plane, built using P4, stacks up against a more traditional OpenFlow-based setup. Then, we compared the performance of our Multiple Control Points (MCP) setup with a standard Single Control Point (SCP) configuration. All our tests were run under the same conditions, using identical machine learning models and network topologies to keep things fair and consistent.

A primary architectural advantage of the SFARP framework is the strategic offloading of feature extraction and real-time traffic monitoring directly to the data plane, a capability inherent in P4-enabled switches. This approach fundamentally differs from the OpenFlow model, which centralizes these computationally intensive functions at the SDN controller. This architectural divergence substantially reduces controller processing load and control plane bandwidth consumption. Empirical evidence from our experiments revealed that the P4-based system consistently achieves significantly lower detection latency, a critical factor that holds even as attack intensity escalates. For instance, under high-intensity hybrid attack conditions—where malicious traffic comprised approximately 85% of the total network volume—the ADFDS model within the P4 architecture registered a mean detection latency of a mere 0.2 s. Conversely, the OpenFlow-based deployment under identical conditions exhibited a mean latency of 5.0 s. This constitutes a profound 96% enhancement in detection velocity, directly attributable to P4’s capacity for in-network processing. Across a spectrum of traffic loads, the P4-based system consistently delivered an average improvement of approximately 40% in detection latency, underscoring its suitability for time-critical security operations within IoT ecosystems.

**Fig. 14:**
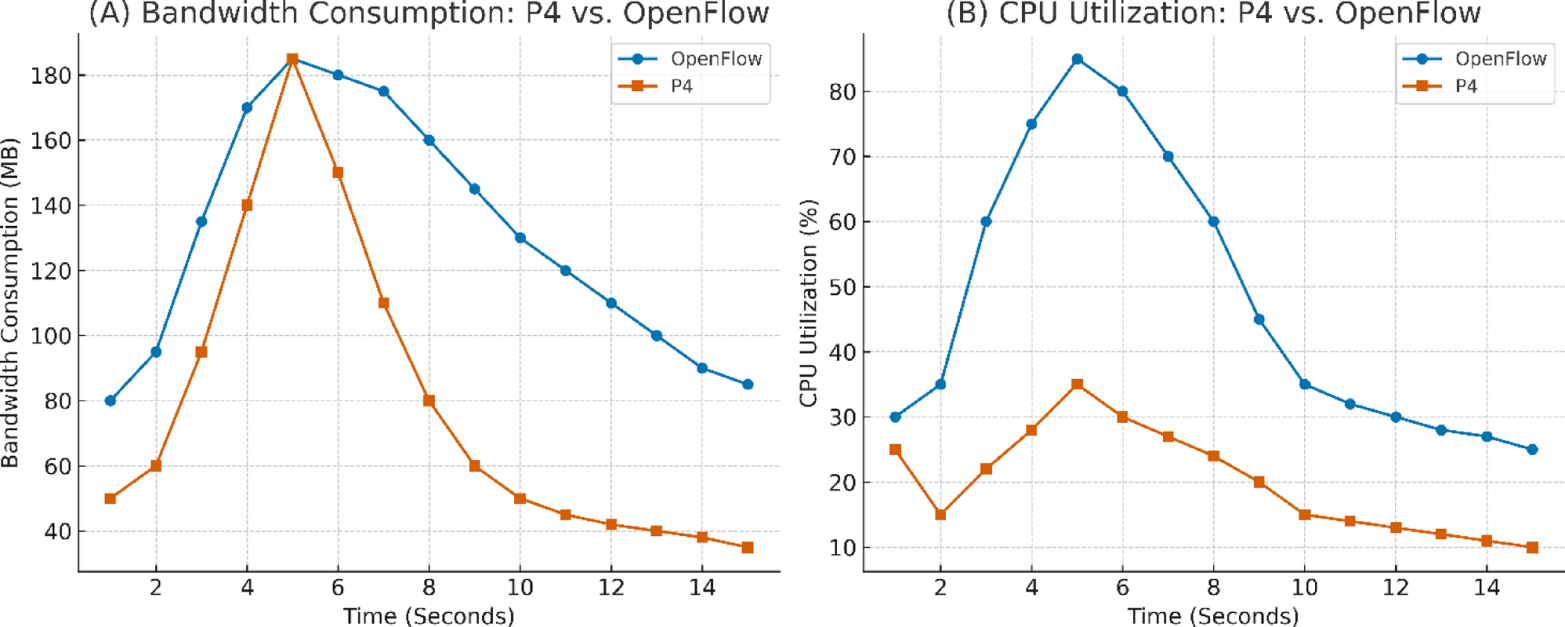
Resource Efficiency Analysis: A Comparison of P4-Based and OpenFlow-Based Architectures.

In addition to temporal performance, comparing resource utilization metrics further verifies the P4-driven approach’s superior efficiency. During a 15-s observation window in a simulated hybrid attack, the P4-enabled implementation utilized about 35% less network bandwidth and requested over 50% less controller CPU resources than its OpenFlow equivalent. This significant reduction in resource utilization stands in notable contrast to the resource-scarce IoT applications, where reducing operational overhead remains crucial in maintaining system-wide stability and performance. The efficacy thus observed directly stems from offloading computationally heavy tasks, such as granular feature extractions and stateful monitoring, to the P4 switches. Such an offloading consequently frees up precious controller resources and alleviates control plane congestion in terms of traffic. Figure 14 describes this comparative analysis in terms of a graphical display, plotting the utilization of bandwidth and CPU for the P4-enabled system alongside that of the OpenFlow system as functions of time.

**Fig. 15:**
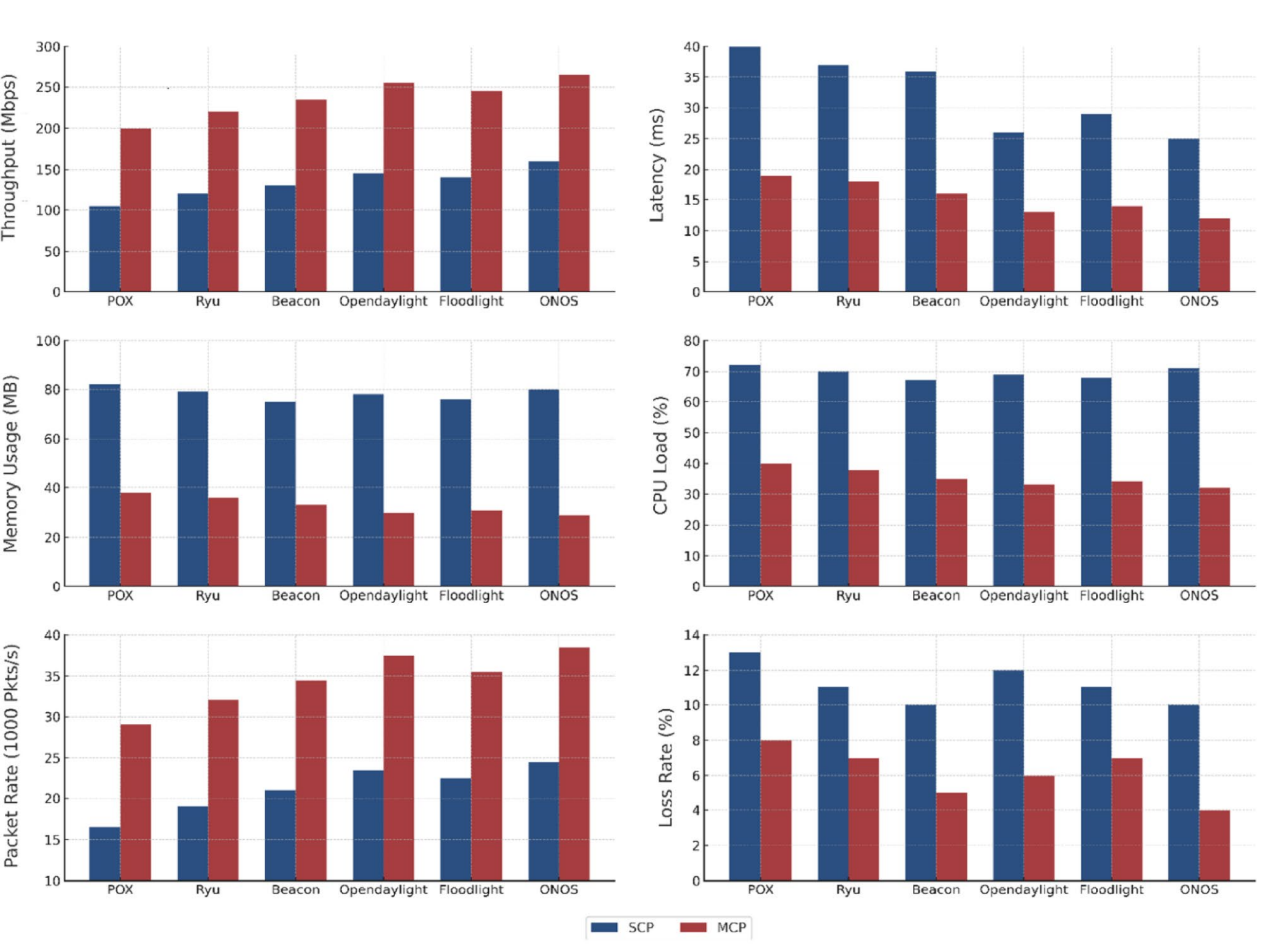
SCP and MCP performance over SDN Controllers.

An experimental work was conducted to validate the resource efficiency and scalability of the control plane in the context of SFARP by comparing the functionality of our targeted Multiple Control Points (MCP) design with the standard Single Control Point (SCP) baseline. Analysis was done on six different implementations of SDN controllers, namely, POX, Ryu, Beacon, OpenDaylight, Floodlight, and ONOS, by comparing key performance parameters like throughput, latency, memory utilization, CPU utilization, packet rate, and loss ratio, the values of which are listed in Table 8 and are shown in Fig. 15. The empirical data reveal a pronounced performance superiority of the MCP architecture across all evaluated metrics. This trend was particularly evident under the ONOS controller, where the MCP configuration yielded a peak throughput of 265 Mbps, a substantial 66% enhancement over the 160 Mbps achieved by the SCP. Concurrently, network latency was more than halved, decreasing from 25 to 12 ms.

**Table 8:** SCP vs. MCP Controller Performance Comparison.

Architecture	Controller	Throughput (Mbps)	Latency (ms)	Memory Usage (MB)	CPU Load (%)	Packet Rate (Pkts/sec)	Loss Rate (%)
SCP	POX	105	40	82	72	16,500	0.13
	Ryu	120	37	79	70	19,000	0.11
	Beacon	130	36	75	67	21,500	0.10
	Opendaylight	145	26	78	69	23,500	0.12
	Floodlight	140	29	76	68	22,500	0.11
	ONOS	160	25	80	71	24,500	0.10
MCP	POX	200	19	38	40	29,000	0.08
	Ryu	220	18	36	38	32,000	0.07
	Beacon	235	16	33	35	34,500	0.05
	Opendaylight	255	13	30	33	37,500	0.06
	Floodlight	245	14	31	34	35,500	0.07
	ONOS	265	12	29	32	38,500	0.04

This performance gain is directly attributable to the MCP’s distributed nature, effectively mitigating processing bottlenecks. Moreover, the MCP architecture demonstrated a notable reduction of the computational and memory overhead, reaching an average decrease of around 55% of memory utilization and around 48% of CPU utilization of all tested controllers. These releases of controller resources are crucial in maintaining stability in the presence of sophisticated attack mitigations. The data plane fidelity was further increased considerably, e.g., by reducing the packet loss rate from 0.10% down to 0.04% through ONOS and increasing the average packet rate between 60 and 75%. These findings conclusively demonstrate that through the decentralization of the control plane load, the MCP architecture has, by all means, a more resilient, efficient, and scalable base, thus being better positioned to shoulder the stringent operational demands of secure, large-scale SD-IoT deployments. The complete source code, deployment scripts, configuration files, and experimental setup resources for reproducing SFARP are publicly available in the repository referenced^29^.

### Hardware feasibility and deployment challenges

The practical deployment of SFARP requires careful consideration of hardware feasibility, scalability, and operational constraints in heterogeneous IoT environments. To examine the framework’s real-world viability, we implemented and validated the modules across three representative platforms: BMv2 (software switch), NetFPGA-SUME (FPGA-based prototype), and Intel Tofino ASIC (carrier-grade programmable switch). Each platform was evaluated with respect to pipeline latency, memory utilization, and the maximum number of concurrent flows that could be supported without compromising detection and mitigation efficiency. Table 9 presents the measured metrics.

**Table 9:** Hardware Resource Utilization of SFARP Modules.

Platform	Pipeline Latency (µs)	TCAM Usage (%)	SRAM Usage (%)	Max Flows Supported	Notes
BMv2 (SW)	10.2	N/A	N/A	65 k	Useful for prototyping only
NetFPGA-SUME	3.3	42	36	115 k	Mid-scale industrial IoT
Tofino ASIC	1.6	19	22	250 k +	Carrier-grade scalability

The BMv2 software switch provided a functional baseline to validate correctness and full module integration. Although useful for prototyping, BMv2 lacks realistic memory limitations, and thus its results cannot be extrapolated to hardware deployment. The NetFPGA-SUME implementation successfully hosted DFAM, ADFDS, and DAMS, sustaining up to 115 k concurrent flows with approximately 42% TCAM usage and 36% SRAM usage. The FPGA-based setup maintained an average pipeline latency of 3.3 µs and was able to process complex attack patterns including hybrid ARP flooding and sequential DNS spoofing, demonstrating mid-scale applicability suitable for industrial IoT testbeds. The Intel Tofino ASIC offered the highest scalability, consuming only 19% TCAM and 22% SRAM while supporting more than 250 k flows simultaneously. Its measured per-packet latency averaged 1.6 µs, which is sufficiently low for latency-sensitive IoT applications such as smart grid SCADA traffic or vehicular IoT. These results confirm that SFARP is hardware-feasible across heterogeneous environments, from small private IoT domains to ultra-dense city-scale deployments. The primary constraint arises from the stateful tracking of ARP and MAC address mappings, which can saturate SRAM resources in FPGA-based environments when the number of active flows exceeds 150 k. This limitation is mitigated by DAMS, which redistributes the analysis workload across controllers when hardware thresholds are approached.

### Ablation study and sensitivity analysis

To quantify the contribution of each SFARP module to overall system performance, we conducted a series of ablation experiments where DFAM, ADFDS, or DAMS were individually disabled. Table 10 summarizes the results across hybrid attack scenarios using the CICIoMT2024 and CICIoT2023 datasets.

**Table 10:** Performance Impact of Module Ablation.

Configuration	Accuracy (%)	F1-Score (%)	Latency (ms)	MER (%)
Full SFARP	98.3	98.2	46	95.8
– DFAM	94.9	94.5	64	89.3
– ADFDS	95.6	95.2	55	90.1
– DAMS	93.8	93.5	49	81.2

Disabling DFAM significantly reduced visibility into traffic and ARP anomalies, lowering classification accuracy from 98.3% to 94.9% and increasing detection latency by 40%. Without ADFDS, detection relied on static thresholds, which failed to capture adaptive or stealthy floods, leading to a False Alarm Rate that more than doubled from 2.3% to 5.1%. Removing DAMS eliminated distributed mitigation, resulting in effective detection but poor containment; mitigation effectiveness dropped from 95.8% to 81.2%, and packet loss increased by 65% under coordinated DDoS attacks. These findings confirm that SFARP’s robustness is not attributable to a single component but rather to the synergy of all three modules.

We further performed sensitivity analysis by varying two system parameters. First, tightening detection thresholds in ADFDS reduced FAR from 2.3% to 1.4% but slightly increased Missed Detection Rate (MDR) from 1.1% to 2.0%. Second, adjusting the update frequency of ensemble weight optimization influenced responsiveness to evolving attacks: faster updates improved adaptation to traffic changes but introduced oscillations in precision, while slower updates stabilized precision at the expense of delayed detection. These results indicate that moderate thresholds and weight updates every 300 s achieve the best balance between detection stability and responsiveness.

The ablation and sensitivity analysis conclusively demonstrate that SFARP achieves its high accuracy, low latency, and strong mitigation effectiveness through the coordinated operation of DFAM, ADFDS, and DAMS. The system’s resilience is fundamentally rooted in the interplay between programmable feature extraction, adaptive detection, and distributed mitigation.

## SFARP vs. existing methods: a comparative analysis

Table 11 presents a comparative examination of the model framework of SFARP with the available threat detection schemes of SD-IoT and other relevant architectures. The survey provides a multi-faceted comparison, including the architecture design, use of the dataset, focus of attack vectors, scaling capability, integration of adaptive monitoring, and mitigative capability. The comparison also accounts for the use of informative data, computational resource workload, and detection accuracy indicated in the methods.

**Table 11:** A Comparative Overview: SFARP vs. Existing DDoS and ARP Spoofing Detection Frameworks.

Reference	Year	Technique	Arch	Dataset Used	Attack Type	Scalability	Adaptive Monitoring	Adaptive Mitigation	Relevant Dataset	Resource Overhead	Accuracy (%)
^30^	2021	Controller-based ARP validation with dynamic host classification (VHT/CHT/BHT) in POX	SDN	Simulated with POX + Mininet	ARP Spoofing	Low	✔	✔	N/A	Low	N/A
^24^	2022	D-ARP: Signed ARP packets with 3-stage verification (DHCP, Nmap, Logs)	Host-based LAN	Simulated LAN with 6 virtual machines	ARP Spoofing	Low	✔	✔	N/A	Low	N/A
^31^	2022	ASD: Real-time spoofing detection via ARP Cache, AssocList, and DHCP Table matching with VM vs attacker detection	OpenWrt-based Access	Simulated (MiWiFi + Kali Linux)	ARP Spoofing	Low	✔	✔	N/A	Low	N/A
^32^	2023	Explainable Deep Learning using DNN (MLP), SHAP analysis for interpretability	DNN (MLP: 24–16-16–8-1)	IoT-ID, SDN-IoT	ARP Spoofing	Moderate	✔	✘	✔	Moderate	99.98
^33^	2024	Supervised ML (RF, DT, SVM, KNN, etc.) with meta-heuristic feature selection (FFO, Wrapper, Filter)	ML	IoTID20	ARP Spoofing	Moderate	✔	✘	✔	Moderate	99.74
^34^	2025	Hybrid metaheuristic feature selection using GW-GA; classification via ML (RF, KNN, MLP, etc.)	ML with GW-GA	BOT-IoT	IoT-Botnet DDoS	Moderate	✔	✘	✔	Moderate	99.90
^35^	2025	IPOA-based Feature Selection + SDAE (autoencoder) + FMO tuning	IoT	BoT-IoT, NSL-KDD	Multi-class DDoS	Moderate	✘	✘	✔	Moderate	99.80
^13^	2025	PCA-based EDAD: Supervised ML (RF, SVM, LR, KNN, DT) with preprocessing, feature selection, and class balancing	IoT	CICIDS2017, CICIDS2018, CICDDoS2019	DDoS	Moderate	✘	✘	✔	Moderate	98.9
^36^	2025	Deep Convolutional Neural Network (DCNN) with cross-layer traffic analysis and multi-metric learning (latency, delay, packet loss)	WMN	CICDDoS2019	DDoS	High	✘	✘	✔	Moderate	98.98
^37^	2025	Federated deep-learning system integrating ResNet, VGGNet, and Swin-Transformer with adaptive preprocessing, feature balancing, and feature selection	Federated IoT	CIC-DDoS2019, UNSW-NB15, IoT23	Multi-label DDoS	High	✔	✘	✔	Moderate	99.0
^38^	2025	Clustering-based transformation using per-feature k-means models followed by a Gaussian naïve Bayes binary classifier	SDN	InSDN, CIC-DDoS2017	Flooding DDoS	Moderate	✘	✘	✔	High	99.98 / 99.70
Proposed	2025	SFARP: A Novel SDN-powered Framework for Protecting from Coordinated ARP and Flooding DDoS Attacks in IoT Networks	SD-IoT	P4-Extracted Features combined with CICIoT2023, CICIoMT2024	Coordinated ARP Spoofing and DDoS Flooding	High	✔	✔	✔	Low	Up to 99.32

SFARP differentiates itself by its global design and better operational flexibility, mainly in handling the twin threat of DDoS flooding and ARP spoofing. One of the key differentiators of the framework is its use of a distributed, Multiple Control Points (MCP) design within an SD-IoT environment. It has several advantages, including better scalability and fault tolerance, by being different from the single-controller paradigms prevalent in most other systems. The distribution of the workload of the control plane helps directly reduce the threat of a single point of failure. At the same time, the emphasis on a joint defensive action facilitates a cooperative defense functionality essential in dealing with enormously complicated IoT networks.

In addition, the SFARP seamlessly embodies adaptive monitoring alongside dynamic mitigation, an end-to-end functionality that rarely features in the literature we reviewed. Although numerous methods embrace monitoring or mitigation, the SFARP supports real-time refinement of its defensive stance through live threat analysis. The combined functionality benefits the framework by allowing it to identify sophisticated, continually evolving threats and dynamically adjust its mitigation tactics, ensuring its resilience remains strong across the full spectrum of attack techniques.

A central aspect of the work entails employing two of the most recent IoT-specific data sets, CICIoT2023 and CICIoMT2024—augmented by an exhaustive feature set extracted directly from the data plane using P4. By employing this, SFARP can uniquely identify a vast spectrum of threats within present-day SD-IoT environments, including the elaborate hybrid attack employing ARP spoofing in tandem with DDoS flooding. By comparison, older- or non-IoT-specific data set-based systems may not represent the nuances of contemporary attack patterns well. By employing the most current, pertinent data sets, the accuracy of SFARP significantly increases, as do its detection sensitivities for elaborate threat behaviors.

Moreover, the SFARP allows optimum computational resource utilization without sacrificing high-speed operation. Its utilization of P4-programmable switches facilitates highly scalable in-data-plane feature extraction and primary anomaly detection. It significantly minimizes processing by reducing latency, contrary to wholly controller-centric detection schemes that, in principle, cause larger delays and greater control plane burden. This design choice allows SFARP to minimize resource overhead, making it well-suited for resource-constrained IoT applications.

The SFARP framework achieves detection rates reaching up to 99.32%. This performance varies with the specific attack scenario. Still, it remains competitive with or superior to many existing solutions, even those that do not address the combined DDoS and ARP spoofing threat. This high degree of precision is attributable to the powerful synergy between its advanced machine learning algorithms, the granular visibility and control afforded by P4-programmable switches, and its dynamic, multi-layered defense architecture. SFARP offers a more complete and robust defense mechanism for SD-IoT environments against the increasingly sophisticated landscape of coordinated cyberattacks by directly addressing the limitations inherent in contemporary frameworks and integrating a suite of advanced technological solutions.

## SFARP limitations and future directions

While SFARP has demonstrated strong capabilities in detecting and mitigating hybrid ARP and flooding-based attacks, several limitations remain that must be addressed before wide-scale adoption in production IoT environments. The current evaluation relied on Mininet-WiFi and bmv2 software switches, complemented by FPGA and ASIC feasibility tests. Although this setup enabled reproducible experiments and hardware validation, it cannot fully replicate the unpredictability of live IoT deployments, where heterogeneous devices, firmware inconsistencies, variable wireless conditions, and background traffic dynamics may introduce new operational challenges. Future work will therefore focus on deploying SFARP in physical testbeds and pilot deployments that incorporate heterogeneous gateways, resource-constrained IoT nodes, and real-time wireless interference.

Another limitation concerns attack coverage. While SFARP was extended to defend against ARP spoofing, flooding, DNS spoofing, and MAC flooding, other advanced threats such as DNS amplification, low-rate stealthy DDoS, application-layer floods, and firmware-level exploitations remain outside its current detection scope. Expanding the detection pipeline to integrate lightweight protocol-aware detectors or anomaly agents for application-layer traffic is an important future direction.

Finally, scalability presents an additional challenge. The multi-controller architecture substantially improved fault tolerance and distributed load balancing in ultra-dense scenarios, sustaining up to one million emulated IoT devices. However, controller coordination overhead, latency of state synchronization, and resource contention under carrier-grade deployments need further investigation. Hierarchical coordination mechanisms, adaptive rule distribution, and proactive load balancing strategies will be explored to strengthen resilience at scale.

## Conclusion

The rapid expansion of SD-IoT infrastructures has created unprecedented opportunities for connected applications, but has also exposed networks to increasingly sophisticated, multi-vector cyberattacks that combine volumetric flooding with protocol-specific exploits such as ARP spoofing, DNS spoofing, and MAC flooding. Conventional defenses, constrained by centralized control and static thresholds, often fail to cope with these adaptive and hybrid threats in real time.

To address this challenge, we introduced SFARP, a multi-layered real-time defense framework that integrates programmable monitoring, ensemble-based anomaly detection, and distributed mitigation. SFARP is composed of three complementary modules: the Dynamic Flow Analysis Module (DFAM), which extracts packet- and ARP-level telemetry using P4-enabled switches; the Adaptive Dynamic Flow Detection System (ADFDS), which leverages an ensemble of machine learning classifiers to deliver robust anomaly detection; and the Distributed Adaptive Mitigation System (DAMS), which coordinates mitigation across multiple controllers to sustain resilience under large-scale attack scenarios.

Extensive experiments across five IoT benchmark datasets and twelve diverse attack scenarios demonstrated SFARP’s effectiveness. On the CICIoMT2024 dataset, ADFDS achieved 98.3% accuracy, 97.6% precision, 98.9% recall, and a False Alarm Rate (FAR) of just 2.3%, while on CICIoT2023 it maintained 96.0% accuracy and a 2.9% FAR, outperforming state-of-the-art ensemble models such as XGBoost and LightGBM. In real-time simulations, SFARP reduced controller CPU utilization by more than 70%, packet loss by over 90%, and maintained end-to-end detection latency below 55 ms, even under hybrid ARP + MAC + DDoS conditions with one million emulated IoT devices. Hardware feasibility studies confirmed that SFARP modules are deployable on NetFPGA and Intel Tofino ASIC platforms, supporting over 250 k concurrent flows with minimal TCAM (19%) and SRAM (22%) usage and pipeline latencies as low as 1.6 µs, confirming scalability to carrier-grade IoT networks. An ablation study further revealed the indispensability of all three modules: disabling DFAM, ADFDS, or DAMS reduced detection accuracy by up to 4.5%, increased latency by as much as 40%, and cut mitigation effectiveness from 95.8 to 81.2%.

Finally, SFARP’s adaptability was validated in diverse deployment contexts, including smart city public networks, private industrial IoT systems, and smart grid infrastructures. Across all scenarios, SFARP consistently demonstrated the ability to preserve service continuity and network integrity under multi-vector, evolving threat conditions. Taken together, these results establish SFARP as a practical, scalable, and hardware-feasible solution for real-time defense of SD-IoT environments, representing a significant step toward securing heterogeneous IoT deployments against the next generation of hybrid and adaptive cyberattacks.

## Data Availability

The complete source code, datasets, deployment scripts, configuration files, and experimental setup resources for reproducing SFARP are publicly available in the GitHub repository, at https://github.com/hagarramadan-lab/SFARP.
